# Supplementary material for: Refining the transcriptome of the human malaria parasite Plasmodium falciparum using amplification-free RNA-seq
Source: BMC Genomics. 2020 Jun 8;21:395. doi: 10.1186/s12864-020-06787-5 (PMC7278070; doi:10.1186/s12864-020-06787-5)
Supplement: Supplementary file 1 — Additional file 1: Supplementary Figures. Figure S1. DAFT-seq schematic. Figure S2. PolyA+ vs Random hexamer priming of mRNA in the DAFT-seq protocol. Figure S3. Method for calling UTRs from DAFT-seq coverage data. Figure S4. Comparison of Chappell et al. 5’ UTRs with the Caro et al. study. Figure S5. Comparison of TSSs with the Adjalley et al. study. Figure S6. 5UTR-seq schematic. Figure S7. Method for calling 5’ UTRs with 5UTR-seq data. Figure S8. Alternative 5’ UTR sets. Figure S9. Homopolymer tracts. Figure S10. PacBio reads show the same features of the transcriptome as DAFT-seq data. Figure S11. Occupancy of covalent histone marks, histone variants, heterochromatin protein 1 (HP1) and the BDP1 chromatin reader at 3D7 TSSs. Figure S12. Nucleosome occupancy at 3D7 TSSs. Figure S13. Distinct temporal changes of TSS usage in the IDC (KAHRP). Figure S14. The TSS frequency and shape landscape for 3D7. Figure S15. TSS motifs in introns and exons. Figure S16. Sequence features of sharp and broad promoters. Figure S17. ApiAP2 motif occurrences relative to predicted TSSs and annotated translation start sites in the 3D7 strain. Figure S18. ApiAP2 motifs around the KAHRP gene. Figure S19. Exitrons in PacBio reads. Figure S20. Alternative splicing in PacBio reads. Figure S21. TSS associated non-coding RNAs. Figure S22. Long non-coding RNAs. Figure S23. Alignment of DAFT-seq data to microarray data. Figure S24. ApiAP2 expression in the 3 P. falciparum strains. Figure S25. AP2-G expression in the 3 P. falciparum strains. Figure S26. Amplitude change distributions of all genes in the 3 P. falciparum strains. Figure S27. Coverage-based UTRs for the 3 P. falciparum strains. Figure S28. Analysis of expression patterns of adjacent gene pairs for the 3 P. falciparum strains. Figure S29. A spliced non-coding RNA opposite GDV1. [file 12864_2020_6787_MOESM1_ESM.docx]

**Supplementary figures and text for:**

**Refining the transcriptome of the human malaria parasite *Plasmodium falciparum* using amplification-free RNA-seq**

Lia Chappell^1^, Philipp Ross^2,3^, Lindsey Orchard^2^, Timothy J. Russell^2^, Thomas D. Otto^1,4^,

Matthew Berriman^1^, Julian C. Rayner^1,5^, Manuel Llinás ^2,6*^

^1^ Wellcome Sanger Institute, Wellcome Genome Campus, Cambridge CB10 1SA, United Kingdom

^2^ Department of Biochemistry & Molecular Biology and Huck Center for Malaria Research, Pennsylvania State University, University Park, PA USA 16802

^3^ Current Address: Department of Biochemistry and Molecular Biology,The University of Chicago, Chicago, IL USA 60637

^4^ Current Address: Institute of Infection, Immunity and Inflammation, MVLS, University of Glasgow, Glasgow G12 8TA United Kingdom

^5^ Current Address: Cambridge Institute for Medical Research, University of Cambridge, Cambridge CB2 0XY, United Kingdom

^6^ Department of Chemistry, Pennsylvania State University, University Park, PA USA 16802

*To whom correspondence should be addressed. E-mail: manuel@psu.edu

**This PDF file includes:**

Figures S1-S29

Supplementary text

References

**Supplementary figures**

Figure S1: DAFT-seq schematic

Figure S2: PolyA+ vs Random hexamer priming of mRNA in the DAFT-seq protocol

Figure S3: Method for calling UTRs from DAFT-seq coverage data

Figure S4: Comparison of Chappell et al. 5’ UTRs with the Caro et al. study

Figure S5: Comparison of TSSs with the Adjalley et al. study

Figure S6: 5UTR-seq schematic

Figure S7: Method for calling 5’ UTRs with 5UTR-seq data

Figure S8: Alternative 5’ UTR sets

Figure S9: Homopolymer tracts

Figure S10: PacBio reads show the same features of the transcriptome as DAFT-seq data

Figure S11: Occupancy of covalent histone marks, histone variants, heterochromatin protein 1 (HP1) and the BDP1 chromatin reader at 3D7 TSSs

Figure S12: Nucleosome occupancy at 3D7 TSSs

Figure S13: Distinct temporal changes of TSS usage in the IDC (KAHRP)

Figure S14: The TSS frequency and shape landscape for 3D7

Figure S15: TSS motifs in introns and exons

Figure S16: Sequence features of sharp and broad promoters

Figure S17: ApiAP2 motif occurrences relative to predicted TSSs and annotated translation start sites in the 3D7 strain

Figure S18: ApiAP2 motifs around the KAHRP gene

Figure S19: Exitrons in PacBio reads

Figure S20: Alternative splicing in PacBio reads

Figure S21: TSS associated non-coding RNAs

Figure S22: Long non-coding RNAs

Figure S23: Alignment of DAFT-seq data to microarray data

Figure S24: ApiAP2 expression in the 3 *P. falciparum* strains

Figure S25: AP2-G expression in the 3 *P. falciparum* strains

Figure S26: Amplitude change distributions of all genes in the 3 *P. falciparum* strains

Figure S27: Coverage-based UTRs for the 3 *P. falciparum* strains

Figure S28: Analysis of expression patterns of adjacent gene pairs for the 3 *P. falciparum* strains

Figure S29: A spliced non-coding RNA opposite GDV1


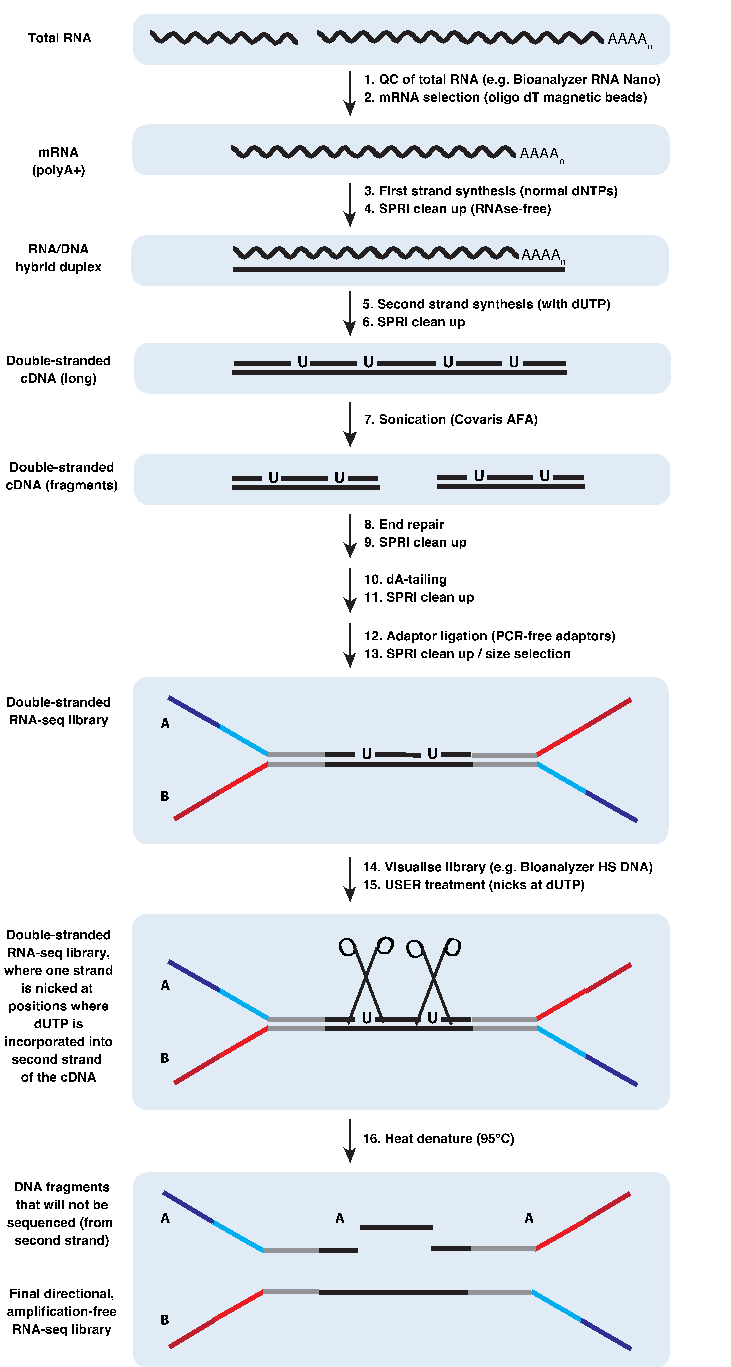


**Figure S1: DAFT-seq schematic**

**Figure S1: DAFT-seq schematic**

Overview of the directional, amplification-free transcriptome sequencing (DAFT-seq) protocol. The input to the protocol is quality-controlled total RNA; poly A+ RNA (mostly mRNA) is then isolated using magnetic oligo (d)T beads. Reverse transcription of full-length mRNA is initiated using oligo d(T) primers, and is followed by a cleanup of the reaction mix using SPRI beads. Next, second strand cDNA is synthesised; dUTP is included instead of dTTP in the dNTP mix to enable retention of directional information. Standard end repair and dA tailing reactions are then performed. Adaptor ligation was modified to include longer “PCR-free” adaptors (similar to those in Kozarewa *et al*. (2009)), which include all the sequence required for Illumina sequencing, allowing the PCR step to be omitted. After SPRI cleanup the second strand cDNA containing dUTP residues is digested using the USER enzyme mix, generating the final directional libraries.


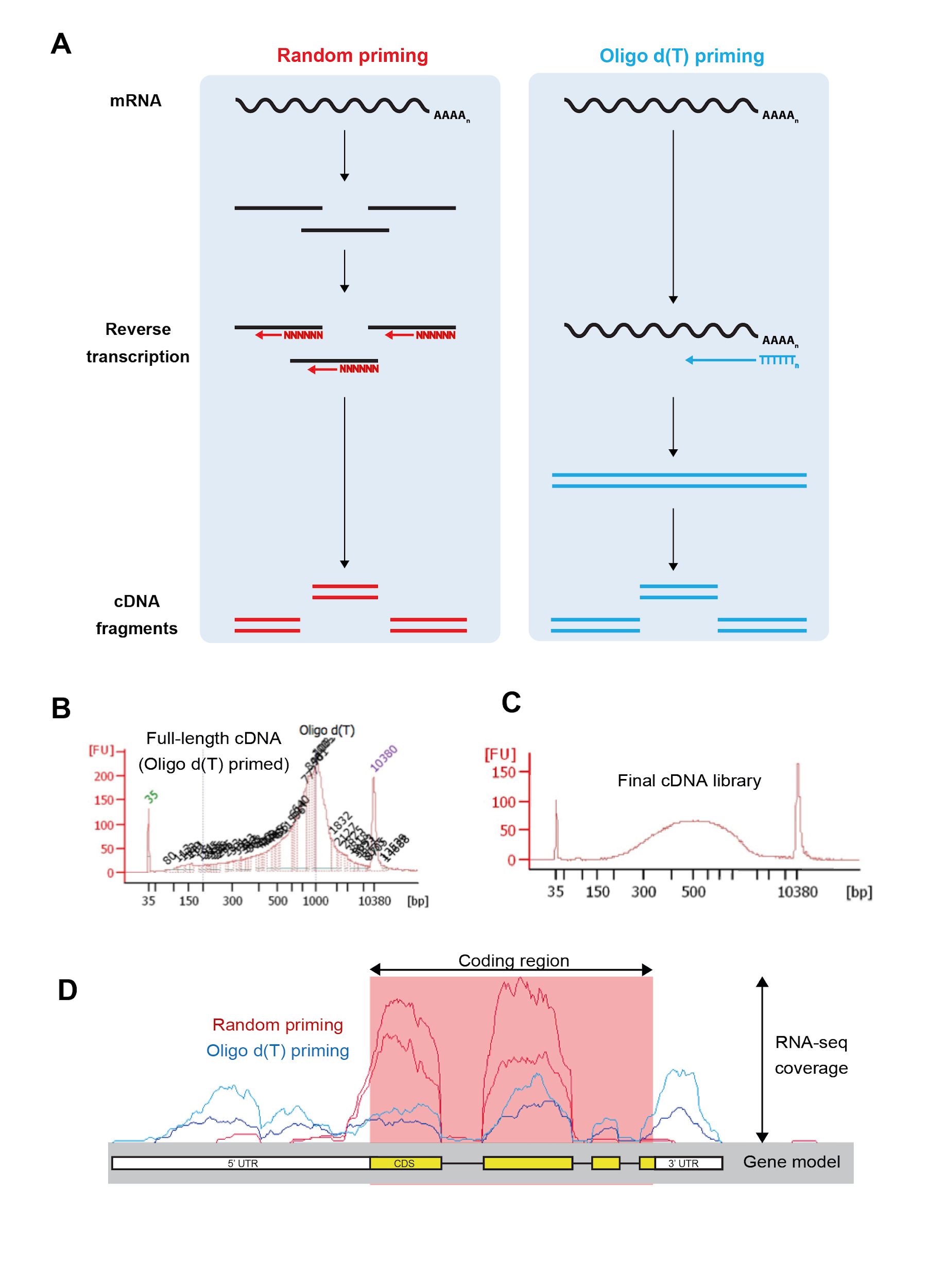


**Figure S2: PolyA+ vs Random hexamer priming of mRNA in the DAFT-seq protocol**

**Figure S2: PolyA+ vs Random hexamer priming of mRNA in the DAFT-seq protocol**

**A)** Left: cDNA synthesis using random priming of mRNA fragments. This is the approach used in most RNA-seq studies, where fragmentation of mRNA is used to improve the evenness of coverage along the length of the transcript. This approach produces uneven coverage in *P. falciparum* RNA-seq libraries, as the mRNA is not primed in AT-rich regions. Right: cDNA synthesis using oligo d(T) priming of full-length mRNA. This approach has previously been shown to have a 3’ end bias in model organisms, but provides more even coverage across genes and in extremely AT-rich regions when used for directional PCR-free *P. falciparum* RNA-seq libraries, as priming only needs to take place once in the polyA+ tail rather than within every AT-rich region.

**B)** Bioanalyzer trace (on HS DNA chip) of full-length cDNA primed using oligo d(T) primers. The peak to the right is >1kb.

**C)** Bioanalyzer trace (on HS DNA chip) of final cDNA library from a DAFT-seq protocol, using cDNA primed with oligo d(T). The average size appears around 500 bp, though the libraries appear larger than the “true” size, due to the longer “Y-shaped” tails on the PCR-free sequencing adaptors.

**D)** RNA-seq coverage traces of PCR-free cDNA libraries made from cDNA primed using random primers only (red trace) or with oligo d(T) primers (blue trace). The libraries were made using the same RNA, so differences in coverage are related to differences in cDNA priming. The random primer library (red trace) has most of its coverage concentrated in the relatively GC-rich coding region of this gene (red box, average AT content of protein-coding sequence is ~80%), with little coverage outside this region (average AT-content of non-coding regions is >90%). Splice sites are seen as sharp drops in coverage within the coding region. The oligo d(T)-primed library shows a flatter coverage distribution across the coding region and adjacent non-coding regions, which are annotated as 5’ and 3’ UTRs. This more even coverage across genomic sequences variable and extreme AT-content enables annotation of UTRs on a genome-wide scale.

|  |  |
| --- | --- |
|  | |
|  | |


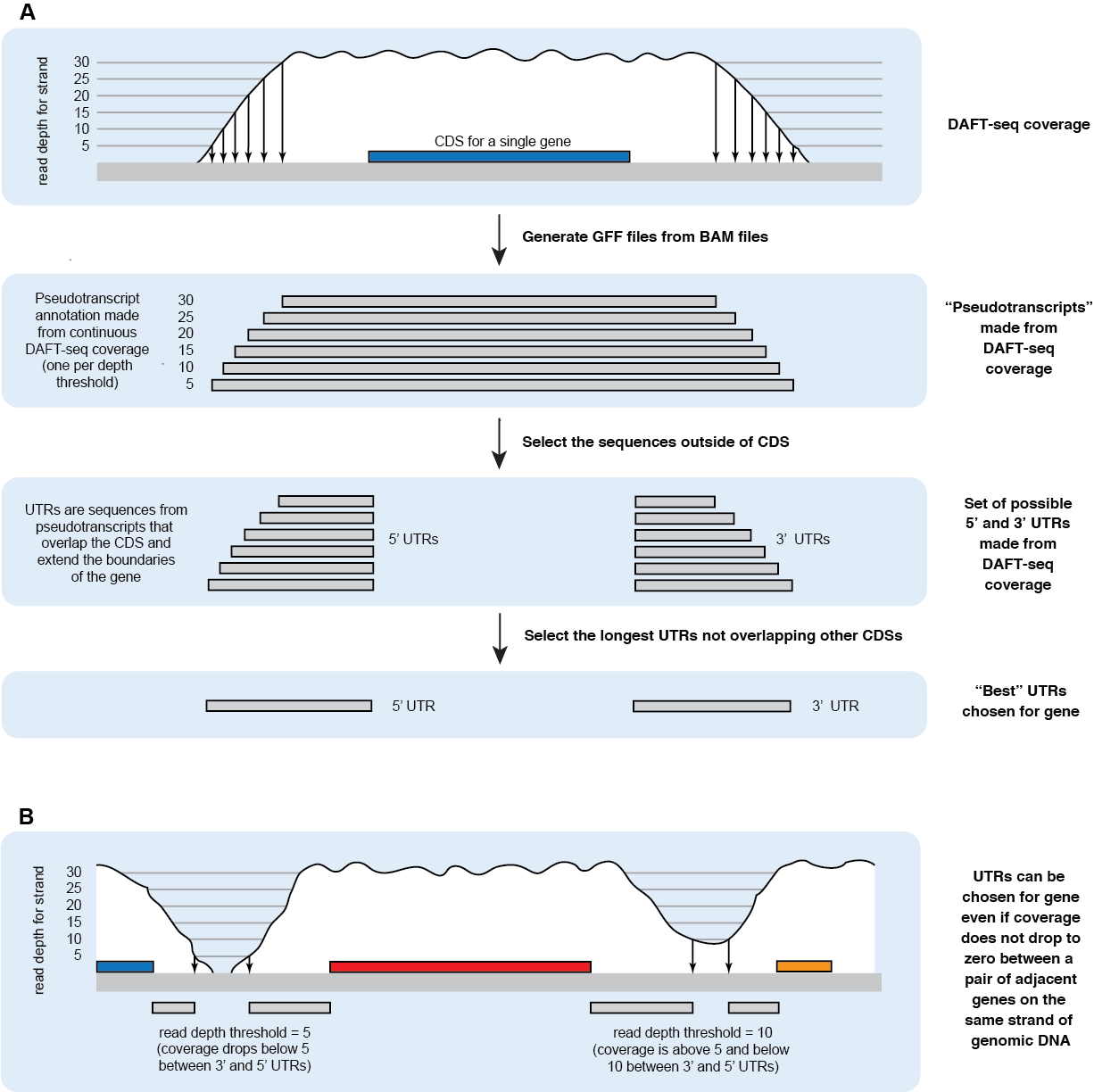


**Figure S3: Method for calling UTRs from DAFT-seq coverage data**

Schematic view of the method for calling 5’ and 3’ UTRs based only on DAFT-seq coverage.

1. Blocks of continuous DAFT-seq data overlapping with each gene model were used to make “pseudotranscripts” at a range of different coverage thresholds (5 to 100 reads depth, in intervals of 5). The coding sequence is “cut out” to generate the 5’ and 3’ UTRs.
2. In cases where adjacent UTRs on the same strand overlap, a more stringent coverage threshold is used to distinguish the two UTRs.

**
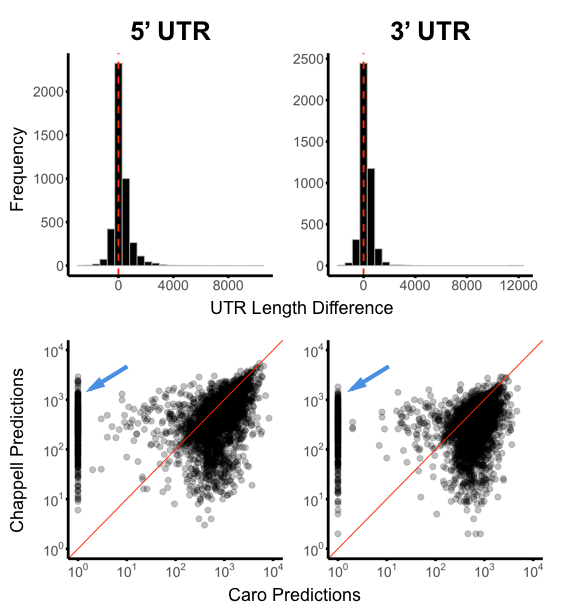
**

**Figure S4: Comparison of UTRs with the Caro *et al.* study**

The 5’ and 3’ UTR predictions from Caro *et al.* were downloaded and compared to 5’ and 3’ UTR predictions in this study. The top row displays the difference in lengths for the whole set, while the bottom row directly compares lengths between individual genes. The longest 5’ and 3’ UTR prediction for each gene from Caro *et al.* were compared to our predictions. Caro *et al.* predictions were on average 230 and 208 bp longer while Pearson correlation estimates were 0.59 (95% CI 0.57,0.61) and 0.36 (95% CI 0.33,0.38) for 5’ and 3’ UTR comparisons, respectively. Notably, despite on average longer UTR lengths, 969 5’ UTRs and 940 3’ UTRs (indicated by the blue arrows) from Caro *et al.* were predicted to have lengths of zero (i.e. transcription begins at the translation start site), but had non-zero length UTRs in our data set. The slope of the solid red line is equal to one.

**
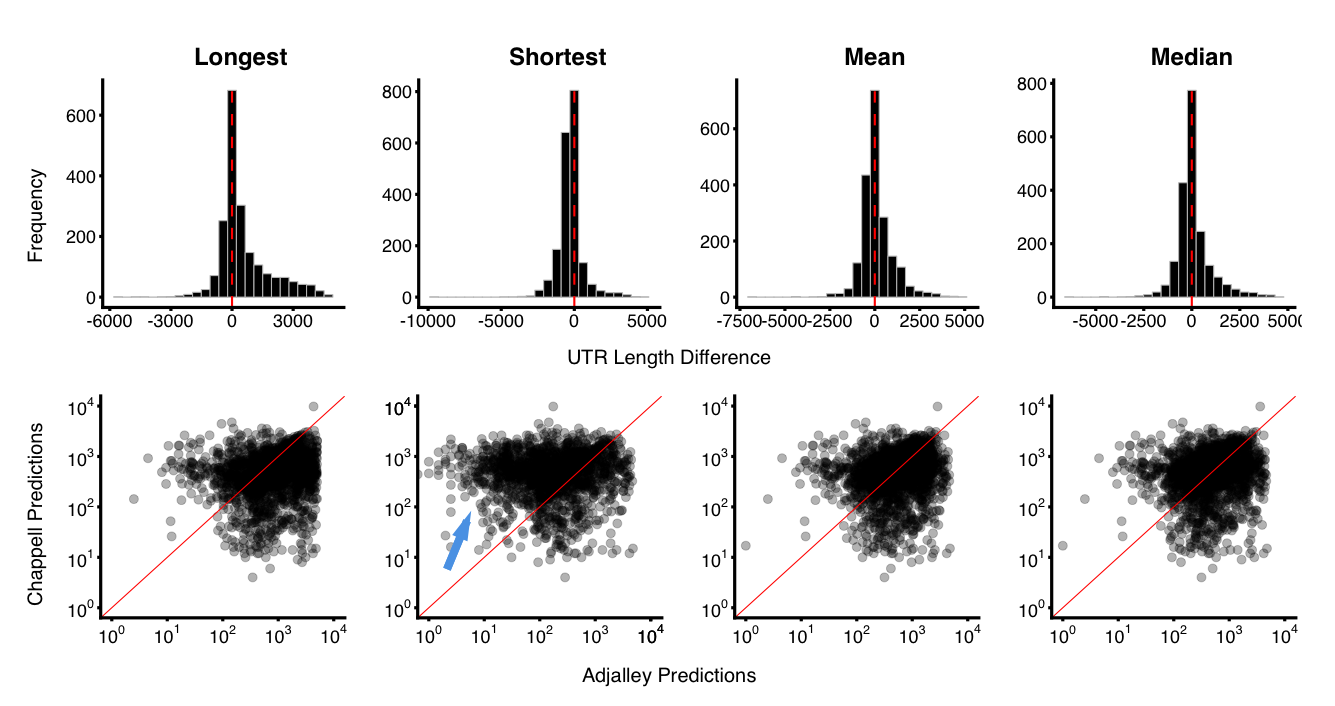
**

**Figure S5: Comparison of TSSs with the Adjalley *et al.* study**

The TSS predictions from Adjalley *et al.* were compared to TSS predictions in this study. Longest, shortest, mean, and median 5’ UTR length predictions for each gene were calculated from TSS predictions in Adjalley *et al.* 5’ UTRs annotated for the same gene were directly compared to 5’ UTR predictions in this study. Predictions were, on average, 562, -263, 117, and 84 bp longer for calculated longest, shortest, mean, and median 5’ UTRs, respectively (minus sign indicates that Chappell *et al.* predictions were, on average, longer). Pearson correlation estimates were 0.05 (95% CI 0-0.9), 0.15 (95% CI 0.10-0.19), 0.14 (95% CI 0.10-0.19), and 0.15 (95% CI 0.10-0.19) for calculated longest, shortest, mean, and median 5’ UTRs respectively. The blue arrow indicates an enrichment of shorter 5’ UTR predictions in the Adjalley *et al.* dataset.

**
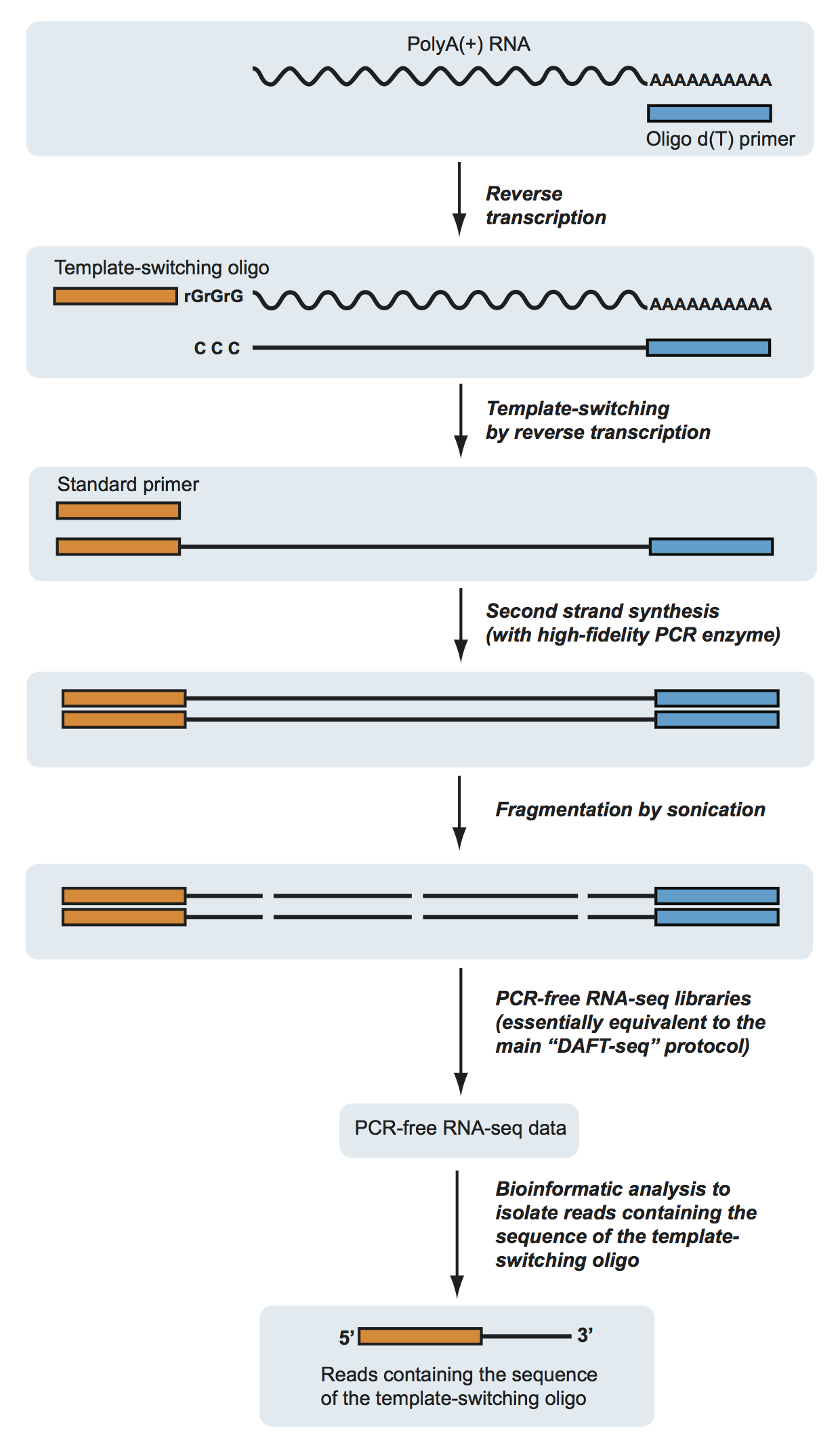
**

**Figure S6: 5UTR-seq schematic**

The 5UTR-seq protocol uses similar molecular biology to the DAFT-seq protocol to generate full-length cDNA from polyA+ mRNA molecules, but additionally includes a template-switching oligo (TSO). A number of modified mouse leukemia virus (MMLV) reverse transcriptases can add non templated Cs to the 3’ end of first strand cDNA where a cap is present at the 5’ end of the mRNA. In the presence of the TSO the reverse transcriptase switches template sequence, the effect of which is to add a common oligo handle to the end of all full-length cDNAs. This TSO “tags” the position in the cDNA that corresponds to the extreme 5’ end of the original mRNA, and can be used to infer the position of the TSS.


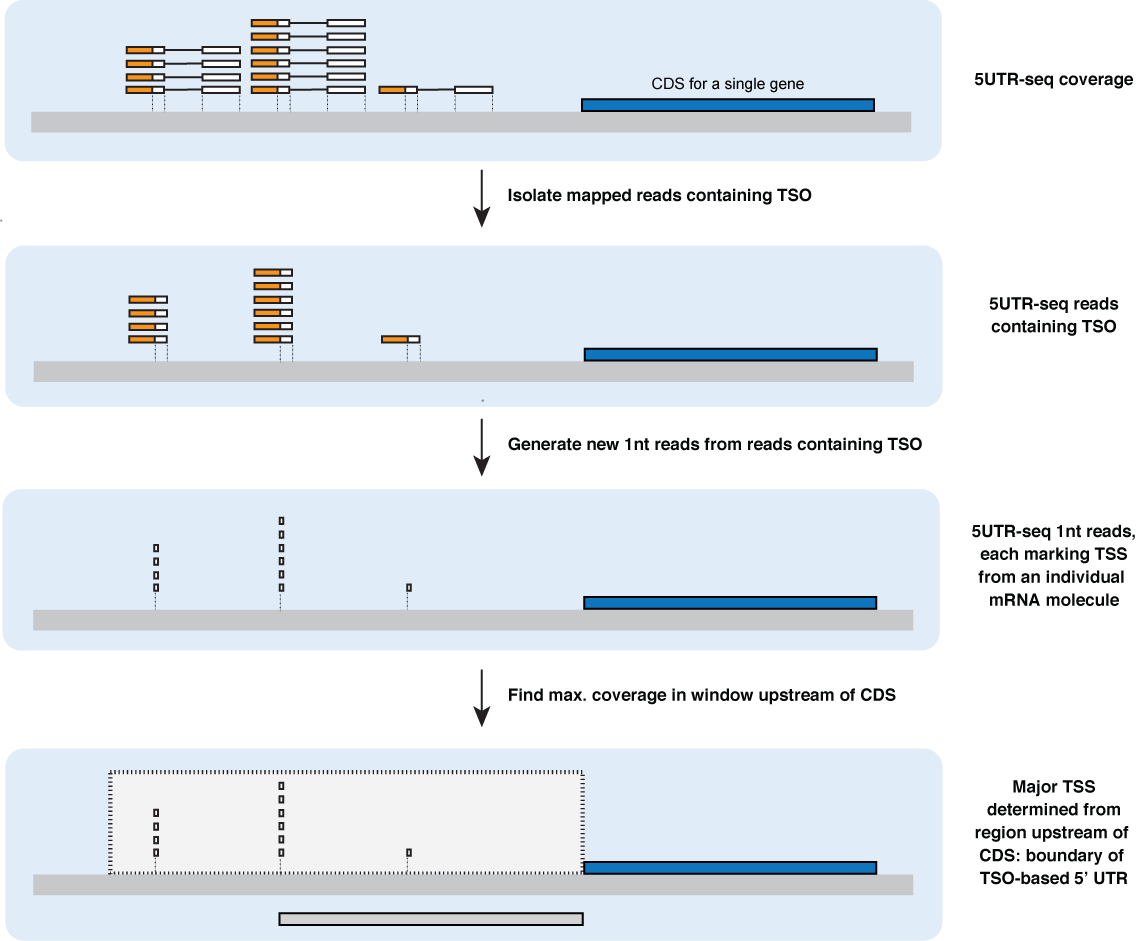


**Figure S7: Method for calling 5’ UTRs with 5UTR-seq data**

The 5UTR-seq reads were mapped to the genome using paired reads, using a mapper (SMALT) that enables “soft-clipping” (overhang) of the TSO, which will not map to the genome. The mapped reads containing the TSO were then isolated (using BLAST), and were processed further to generate single nt coordinates that correspond to the first transcribed bases. For each gene, the reads corresponding to the TSSs of individual molecules were analysed for a defined window upstream of the coding sequence. The relative heights of stacks of reads correspond to relative frequencies of TSS usage.

| **A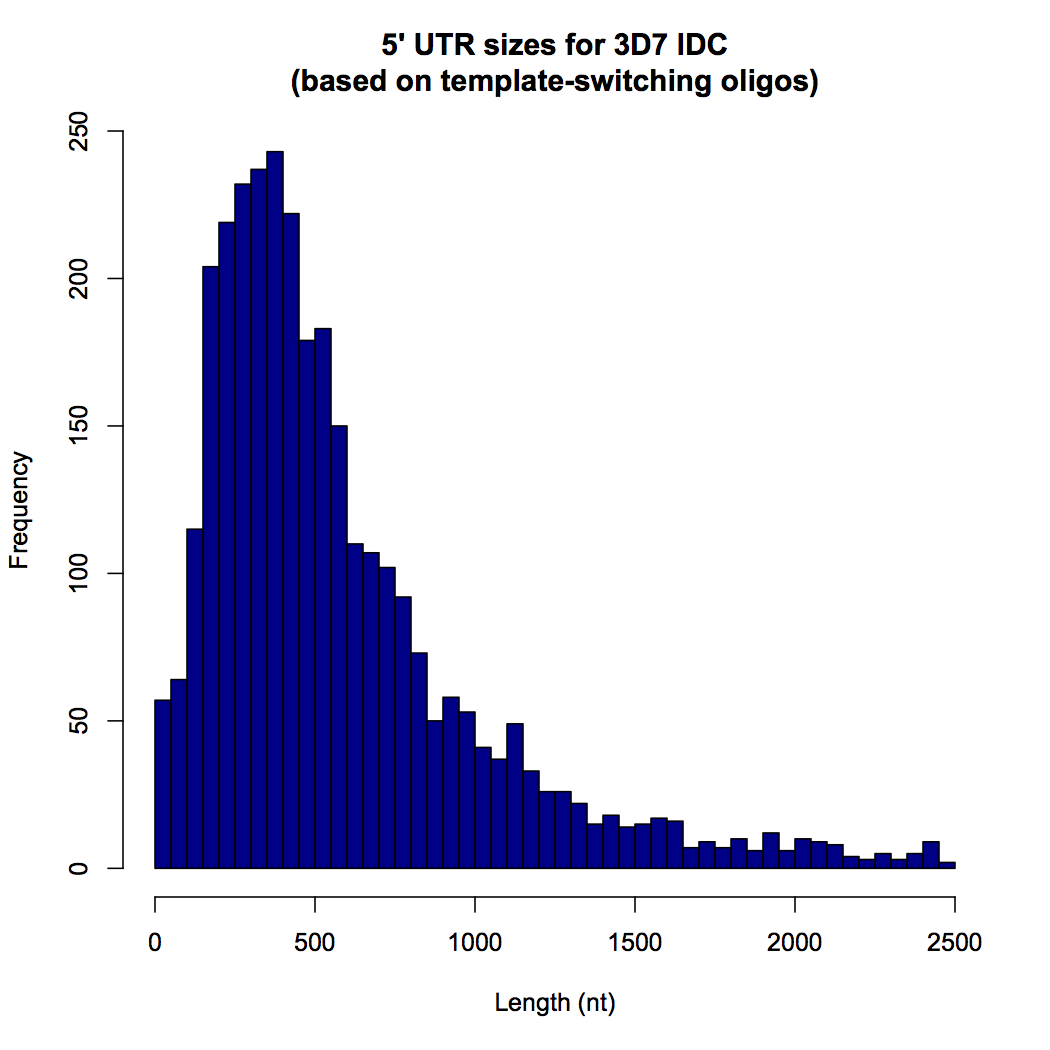** | **B**  **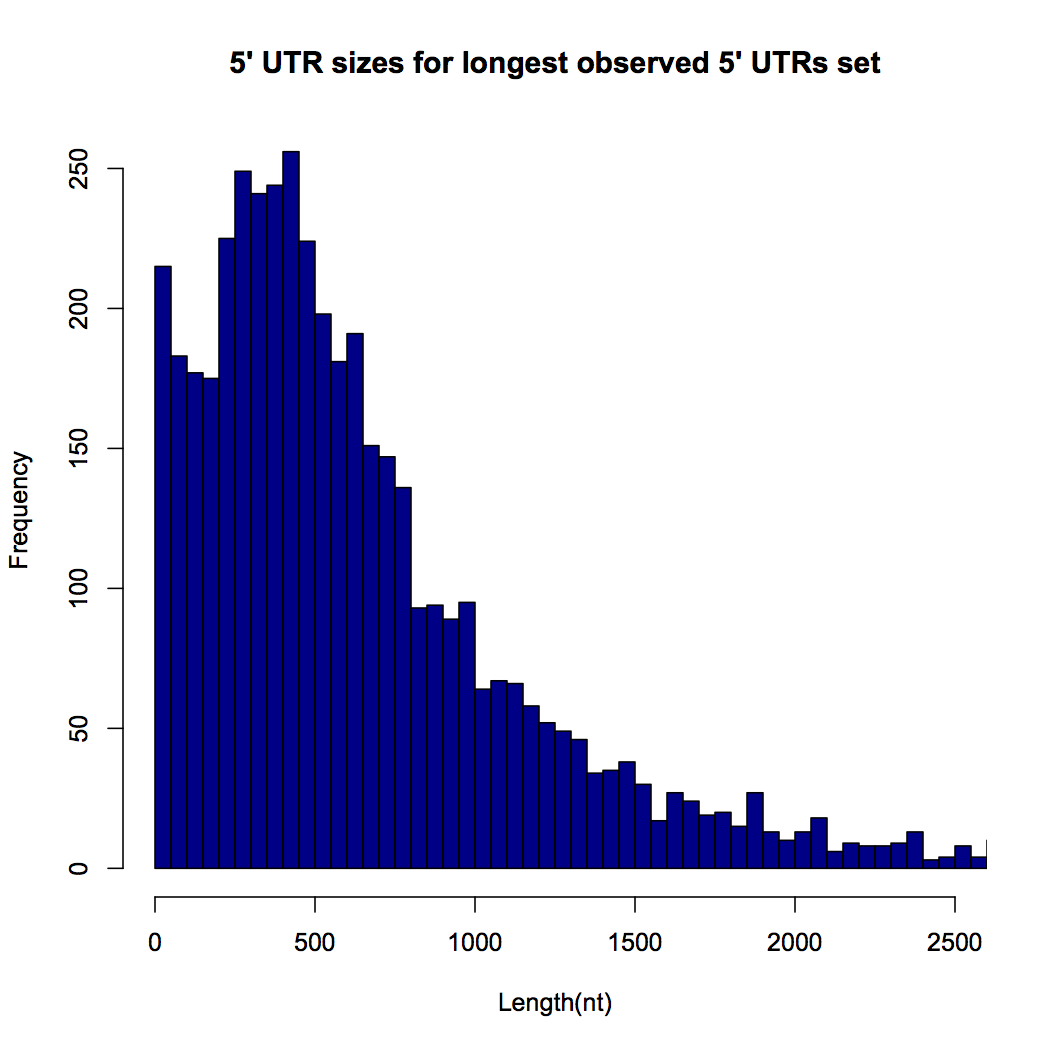** |
| --- | --- |
| **C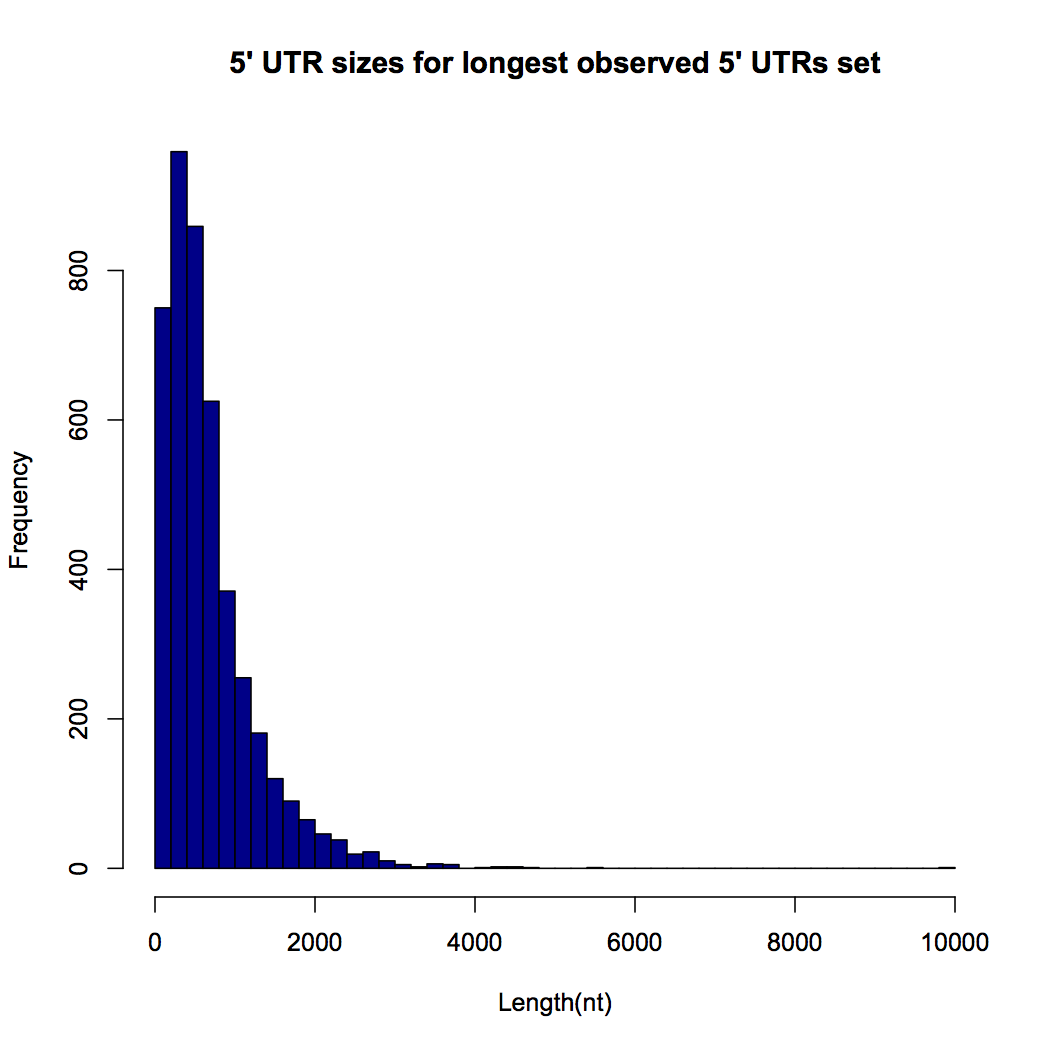** | **D**  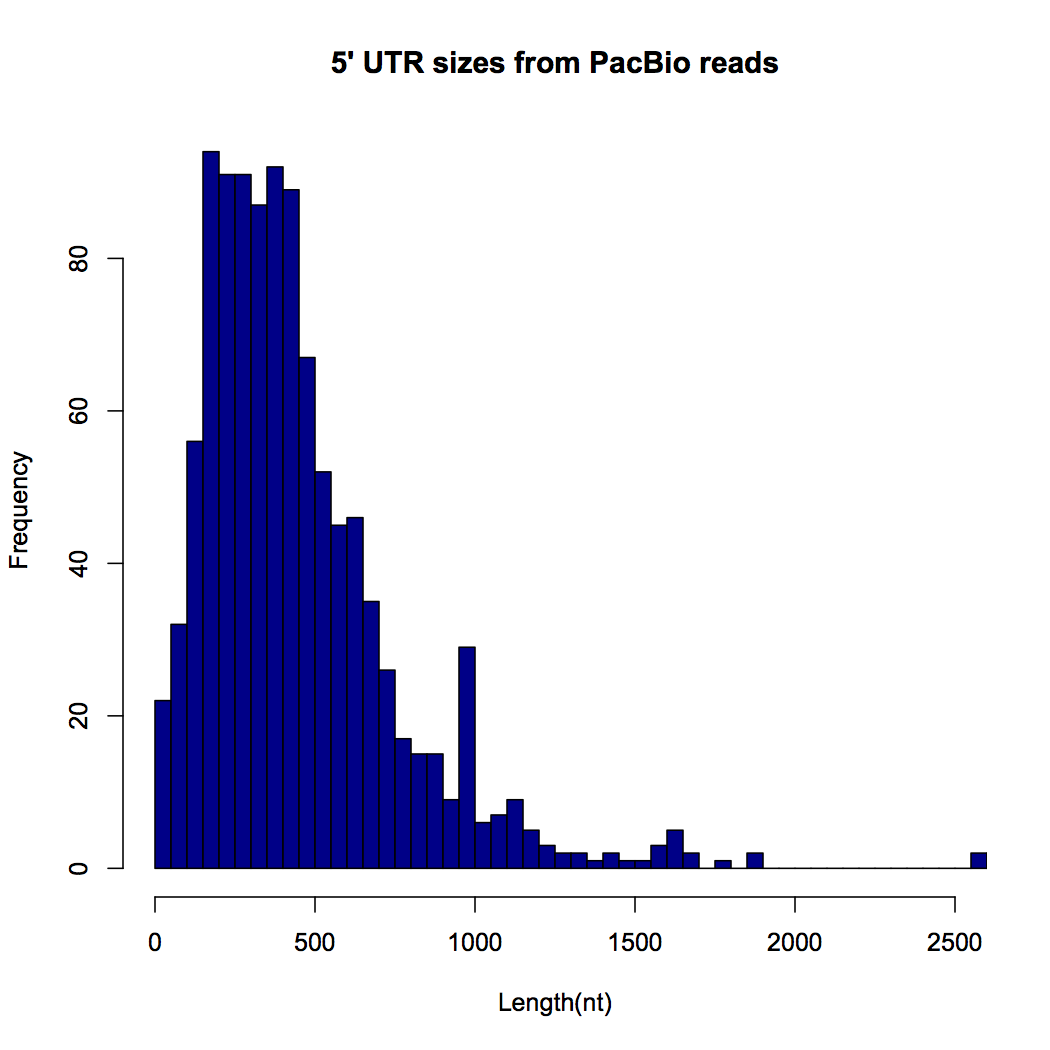 |

**Figure S8: Alternative 5’ UTR sets**

**A)** TSO-based 5’ UTRs (maximum length set to 2,500 nt in detection).

**B)** Longest observed 5’ UTRs from the combined set (scale shown to 2,500 nt).

**C)** Longest observed 5’ UTRs from the combined set (scale shown to 10,000 nt).

**D)** PacBio TSO-based 5’ UTRs (maximum length set to 2,500 nt in detection).

**
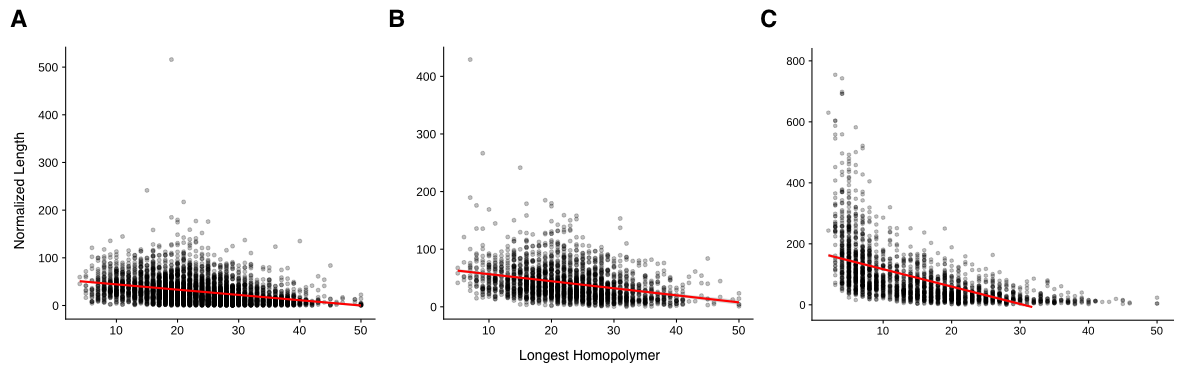
**

**Figure S9: Homopolymer tracts upstream of predicted 5’ UTRs**

1. The 5’ UTR length of all 5’ UTR sequences plus 100 bp ustream, normalized by the longest homopolymer tract plotted as a function of the longest homopolymer tract found within. There is a modest, but significant negative correlation of -0.320 (p-value of 4.10e-56; 95% confidence intervals of -0.357 to -0.283) suggesting that where there are longer homopolymer tracts, the predicted 5’ UTRs are also shorter.
2. The 5’ UTR length of 5’ UTR sequences that were “repaired” plus 100 bps ustream, normalized similar as in A. Again, we see a modest, but significant negative correlation of -0.335 (p-value of 2.33e-115; 95% confidence intervals of -0.361 to -0.308).
3. 100 bp ustream of 5’ UTR sequences that were repaired, normalized similar as in A. We see an even more significant negative correlation of -0.561 (p-value of 5.26e-191; 95% confidence intervals of -0.588 to -0.532). We interpret these results as showing that low complexity regions within *P. falciparum* intergenic regions are responsible for causing gaps in coverage that may bias UTR predictions towards shorter lengths.


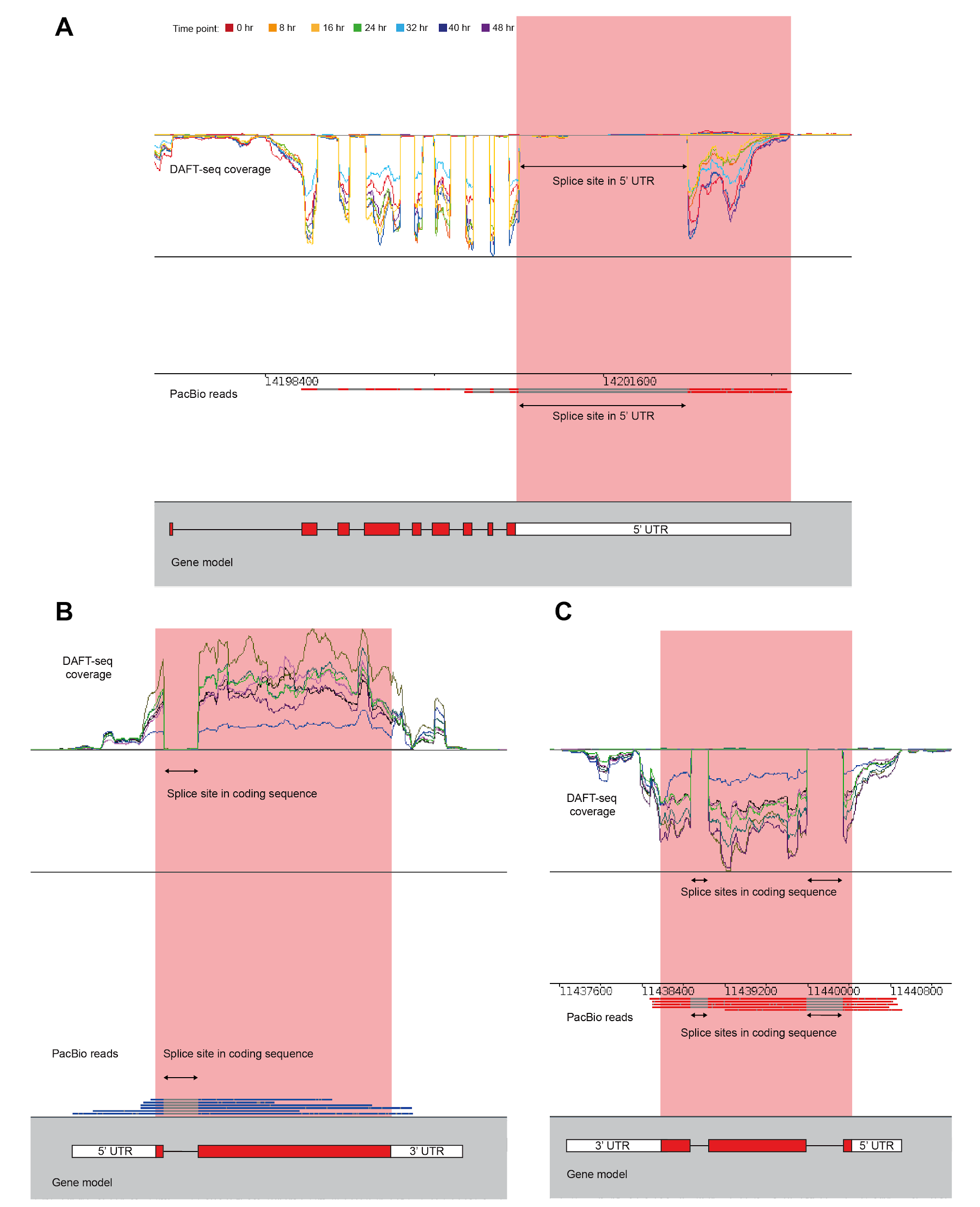


**Figure S10: PacBio reads show the same features of the transcriptome as DAFT-seq data**

**A)** The longest 5 ’UTR (Pf3D7_1136500) in the PacBio data set (middle panel) is very similar to the 5’ UTR of the same gene in the DAFT-seq data set (top panel).

**B)** The splice sites found in the PacBio reads for the gene Pf3D7_0917900 (middle panel) are the same as those found using the DAFT-seq data set (top panel).

**C)** The isoform structure found in the PacBio reads for the gene Pf3D7_1008700 (middle panel) is the same as that found using the DAFT-seq data set (top panel).

**
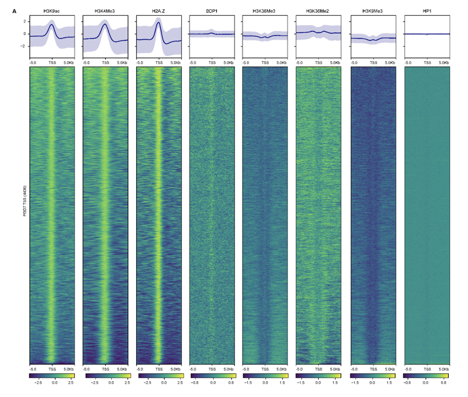
**

**
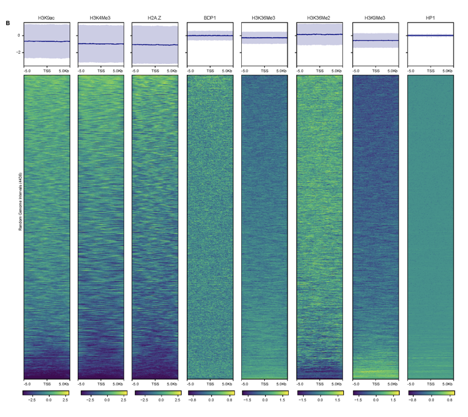
**

**Figure S11: Occupancy of covalent histone marks, histone variants, heterochromatin**

**protein 1 (HP1) and the bromodomain protein 1 (BDP1) chromatin reader at 3D7 TSSs**

**A)** The TSSs identified from TSO-based 5’ UTRs (n=4436) are plotted against the log2 normalized ratio of ChIP enrichment/input for each dataset.

**B)** For comparison, ChIP enrichment at equivalently sized random genomic regions chosen from the same chromosome as the original TSS intervals.

These data illustrate that histone marks and chromatin factors associated with transcriptional activation are highly enriched at TSSs, while repressive marks are not.


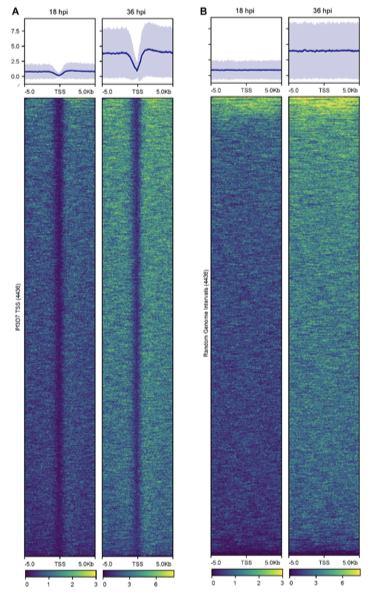


**Figure S12: Nucleosome occupancy at 3D7 TSSs**

**A)** The TSSs identified from TSO-based 5’ UTRs (n=4436) are plotted against nucleosome occupancy at 18 and 36 hours post-infection.

**B)** For comparison, the same data is plotted with respect to equivalently sized randomly chosen intervals from the same chromosome as each TSS.

These data suggest that nucleosome occupancy is well-positioned starting at the TSS at these timepoints.

**
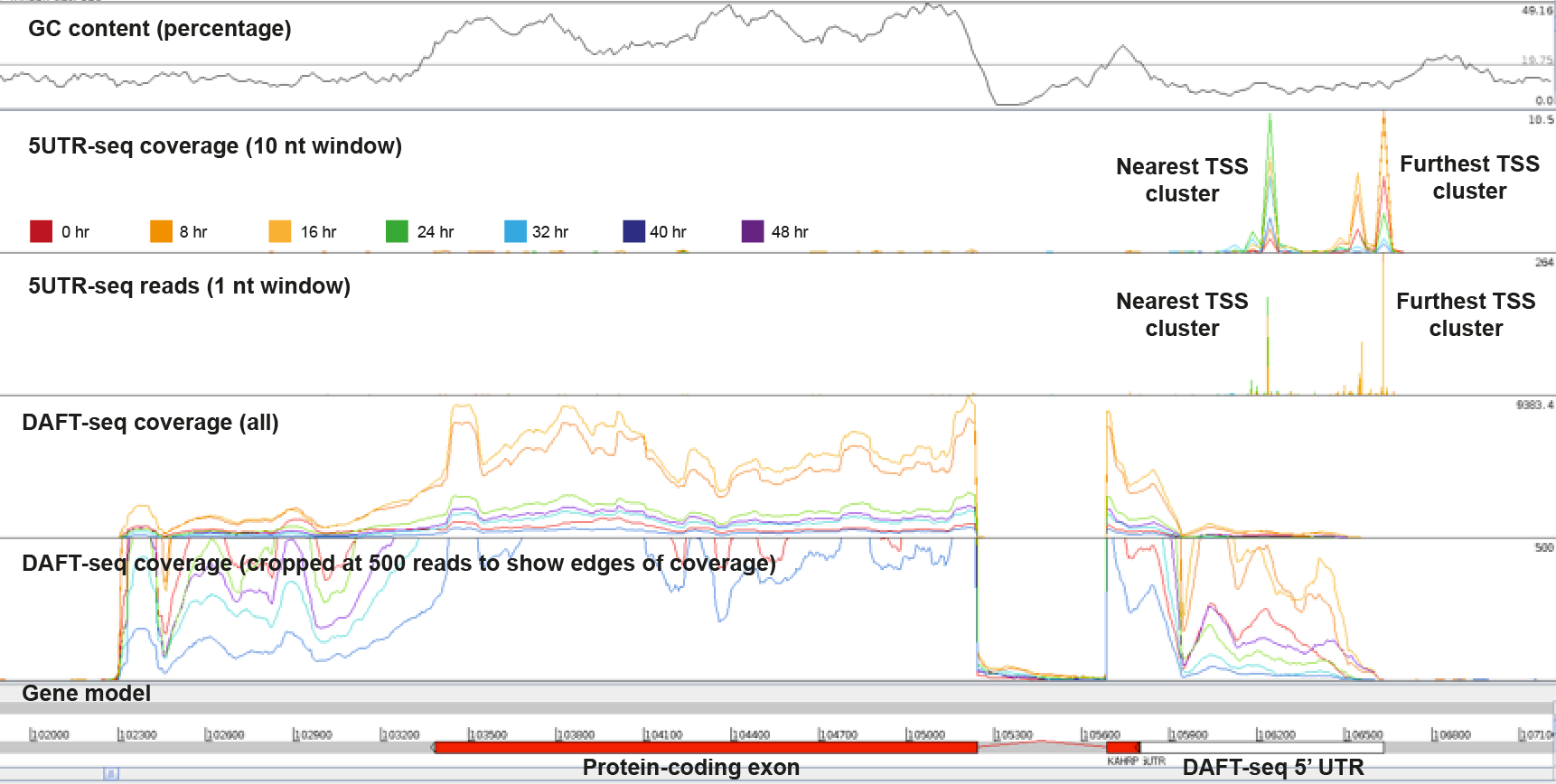
**

**Figure S13: Distinct temporal changes of TSS usage in the IDC (KAHRP)**

There is a change in the relative usage of TSSs between the clusters of TSSs that are nearest and furthest from the start codon of the KAHRP gene between different time points in the IDC (see key for colours of different time points).

**
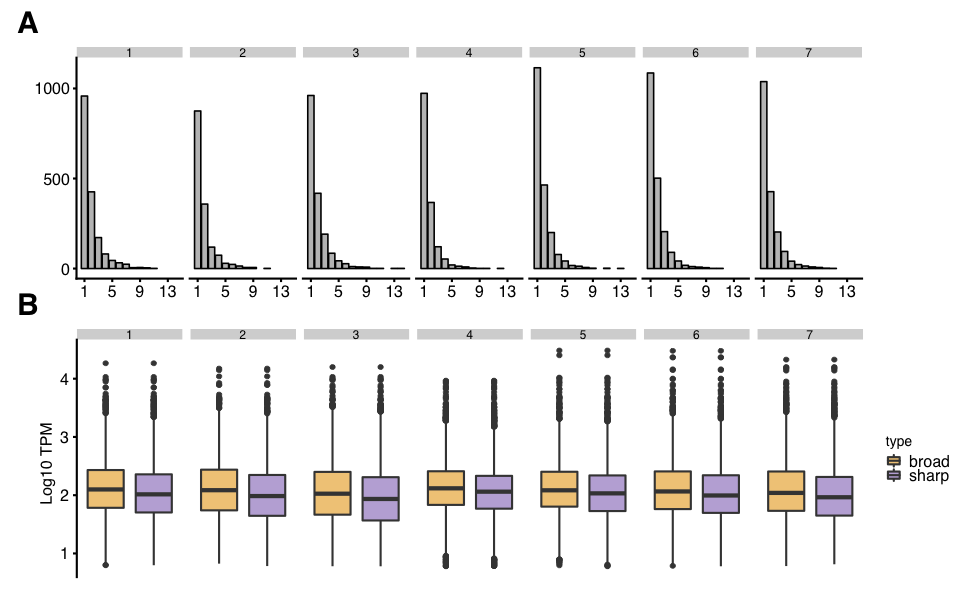
**

**Figure S14: The TSS frequency and shape landscape for 3D7**

**A)** The number of annotated tag clusters (TCs) per gene, per time point (7 panels). Most genes are annotated with just a single TC for each time point (tallest bar,~1000 genes in each time point). However, many genes also have multiple annotated TCs per time point.

**B)** Expression of genes annotated with broad promoters are significantly (Welch Two Sample t-test) and consistently more highly expressed than genes annotated by sharp promoters (p-values <1e-8 for all time points).

**
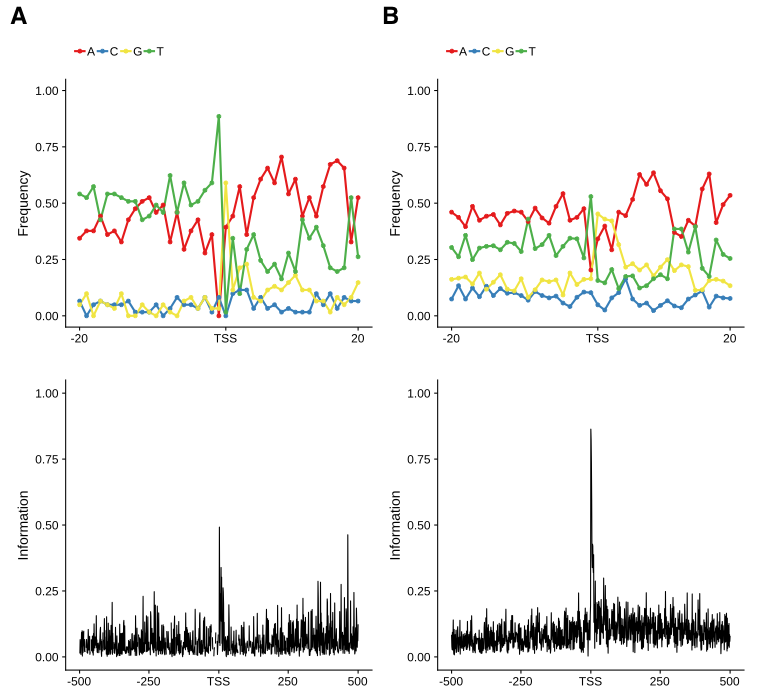
**

**Figure S15: TSS motifs in introns and exons**

**A)** Top panel: average nucleotide frequency for each position surrounding predicted TSSs found within introns (n=61). Lower panel: calculated information content for each position.

**B)** Top panel: average nucleotide frequency for each position surrounding predicted TSSs found within exons (n=389). Lower panel: calculated information content for each position.


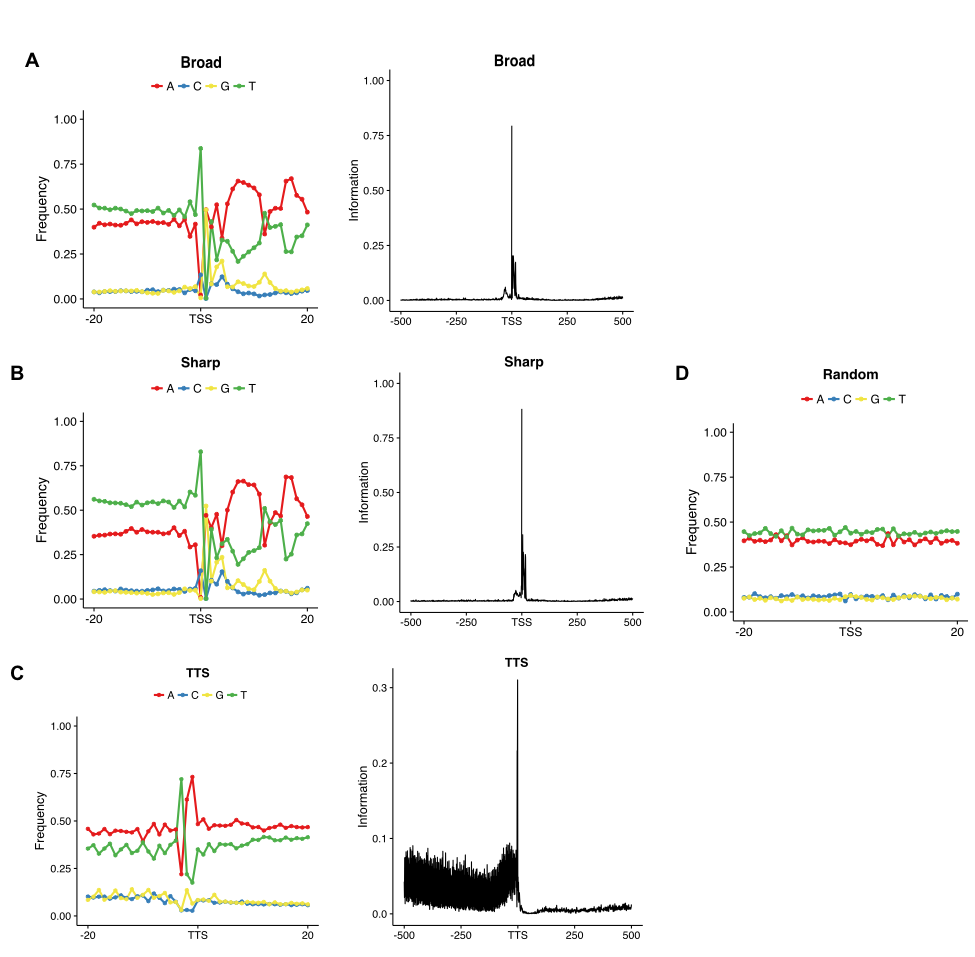


**Figure S16: Sequence features of sharp and broad promoters**

**Figure S16: Sequence features of sharp and broad promoters**

**A)** Left panel: nucleotide frequency surrounding TSSs found in broad promoters, in a 40 bp window centred on the TSS. Broad promoters show distinct sequence patterns surrounding TSSs. A consensus “TG/AG/A” appears surrounding the -1 and +1 nucleotides. Right panel: information content for each position within a 1000 bp window. We observe a peak at defined TSSs and a much smaller peak within 50 bp upstream.

**B)** Left panel: nucleotide frequency surrounding TSSs found in sharp promoters, in a 40 bp window centred on the TSS. Sharp promoters show nearly the same sequence patterns as broad along with a similar consensus sequence.content for each position within a 1000 bp window. Right panel: information content for each position within a 1000 bp window. As with broad promoters, this shows a peak at defined TSSs and a much smaller peak within 50 bp upstream.

**C)** Left panel: nucleotide frequency surrounding transcription termination sites (TTSs). TTSs have subtle, but distinguishable sequence patterns.Right panel: information content for each position within a 1000 bp window, which shows a major change in variability following the termination site.

**D)** Randomly chosen sites throughout the genome show a uniform distribution of nucleotide frequencies.

**
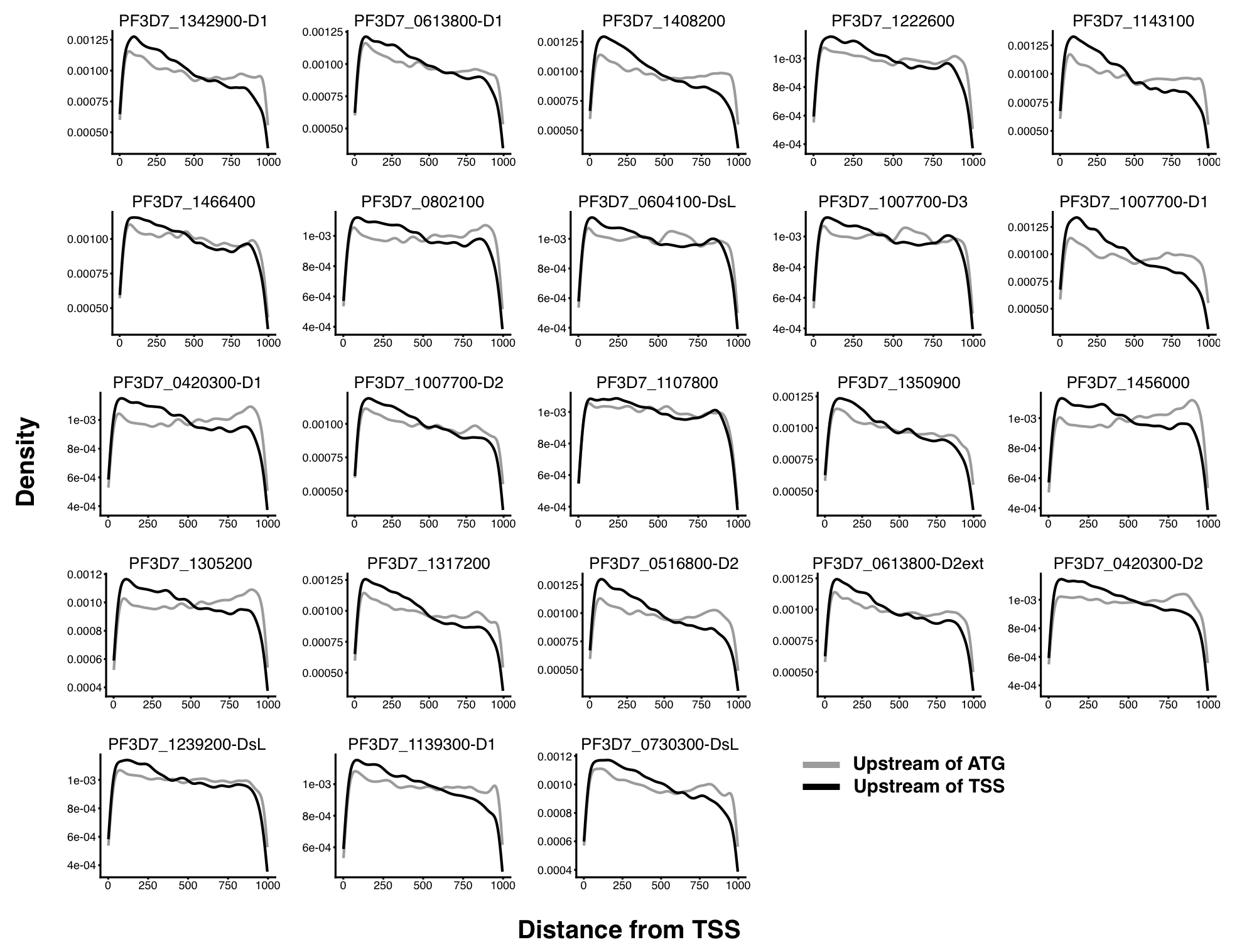
**

**Figure S17: ApiAP2 motif occurrences relative to predicted TSSs and annotated translation start sites in the 3D7 strain**

Motif occurrence density plots from the start (0 bp upstream) to end (1000 bp upstream) of sequences upstream of either predicted TSSs or annotated and highly curated translation start sites (ATG). We observe a consistent increase in frequency within 0–250 bp of predicted TSSs in 3D7 relative to sequences upstream of ATGs. The 3D7 strain is representative of a general trend seen in all strains.

**
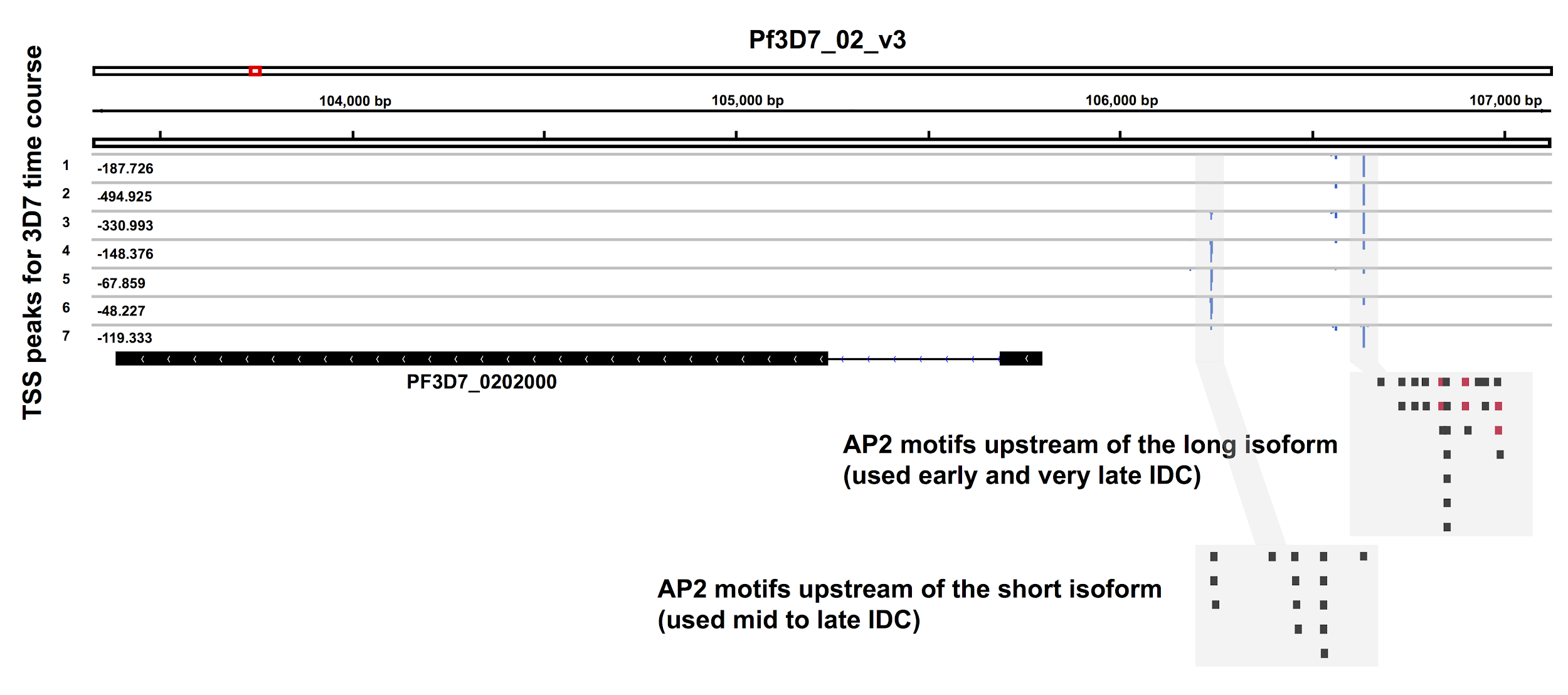
**

**Figure S18: ApiAP2 motifs upstream of the annotated KAHRP translation start site**

The TSS landscape of the knob-assisted histidine-rich protein (KAHRP) shows a dynamic pattern throughout the IDC and exhibits a long and short isoform of its 5’ UTR. Different motif occurrences found on both strands are displayed for long and short isoforms. Motifs highlighted in red show the binding sites of the AP2 transcription factor encoded by the gene *Pf3D7_1466400* (*Pf14_0633)*: these motifs are only found upstream of the long isoform.

**
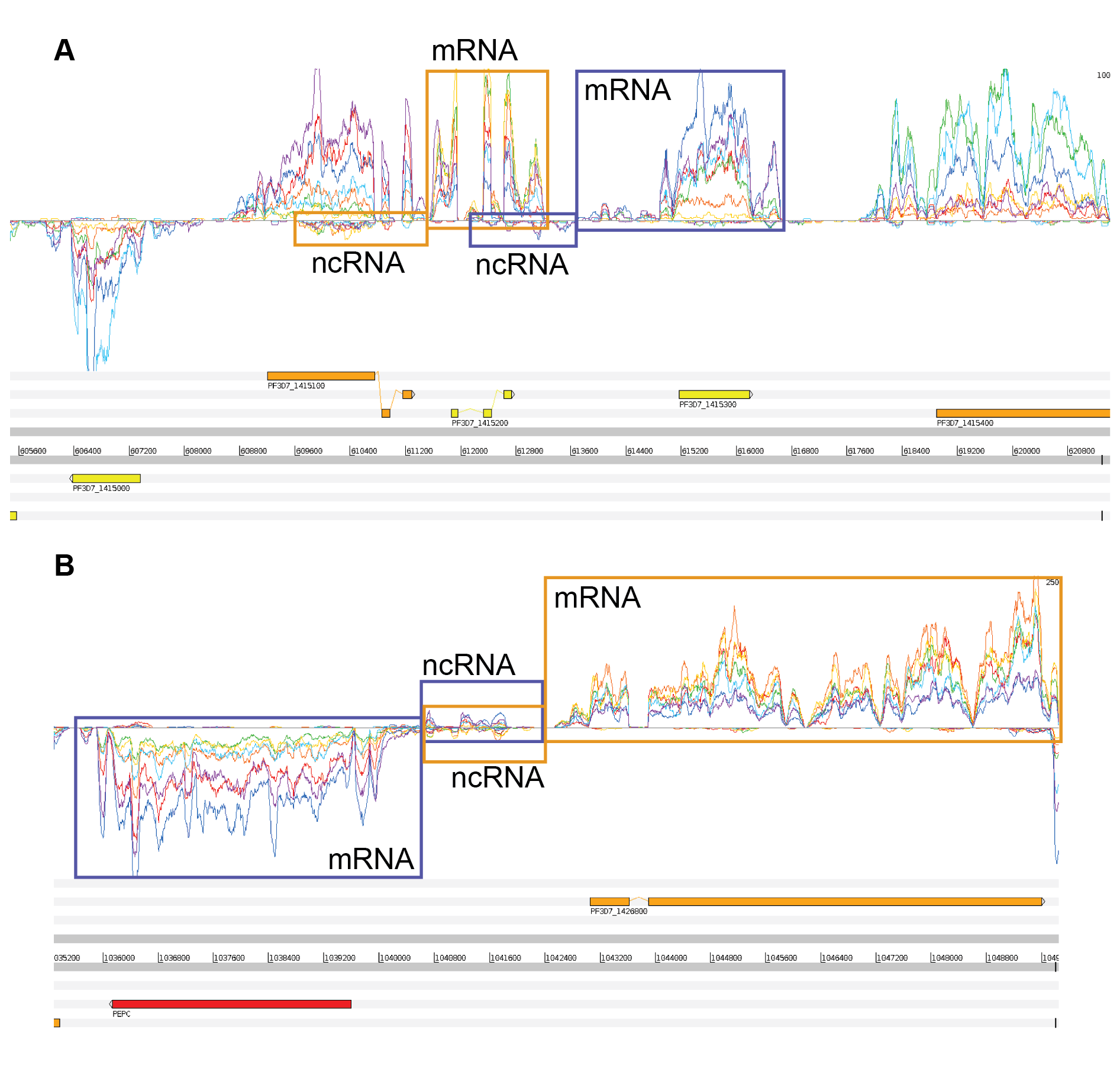
**

**Figure S19: TSS-associated non-coding RNAs**

Pairs of mRNA and correlated antisense non-coding RNAs can be found in a number of genomic locations, additionally to the example shown in figure 4. In panel A, one of the ncRNAs (in the purple box, bottom strand) is antisense to an upstream mRNA (orange box, top strand). In panel B, two of these ncRNAs are in antisense orientations relative to each other- information from the time enables correlated patterns of expression to define the transcript pairs.

**
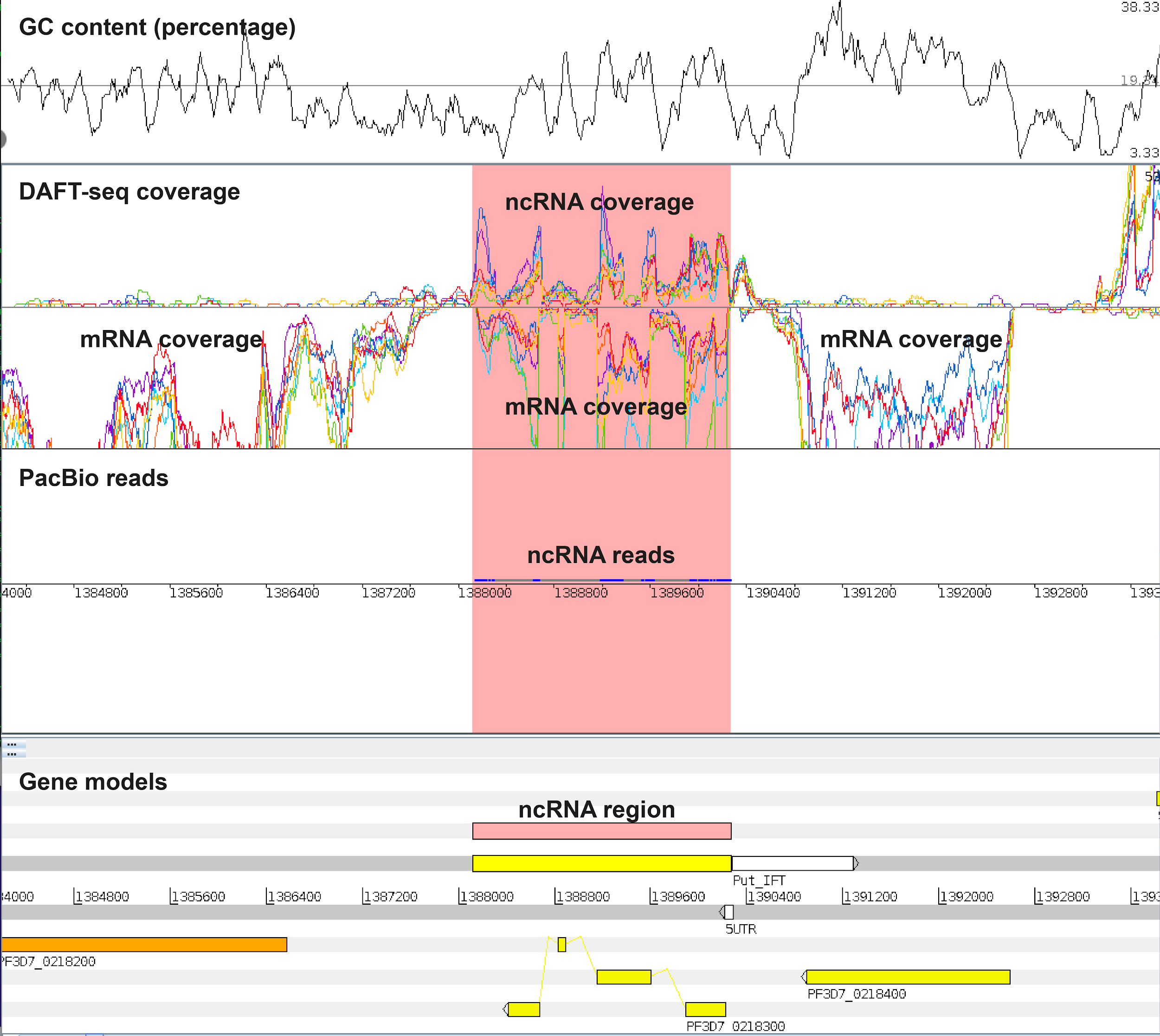
**

**Figure S20: A long non-coding RNA**

A non-coding RNA (present within the region highlighted in red) can be identified opposite the gene PF3D7_0218300 in both DAFT-seq and PacBio data.

**
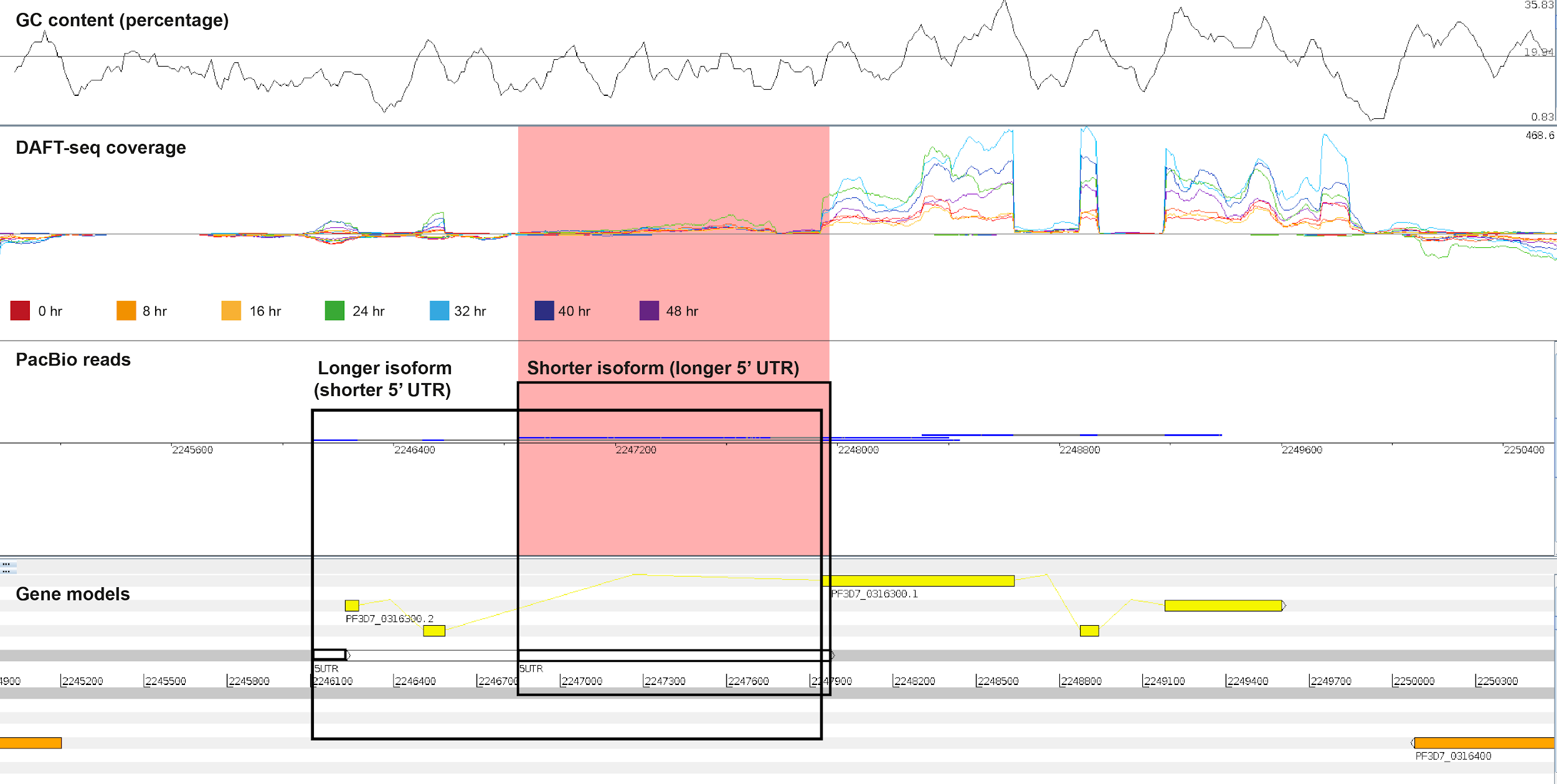
**

**Figure S21: Alternative splicing in PacBio reads**

Two alternative isoforms of the gene PF3D7_0316300 are captured in PacBio reads, where the reads capture different TSSs and different use of splice sites.

**
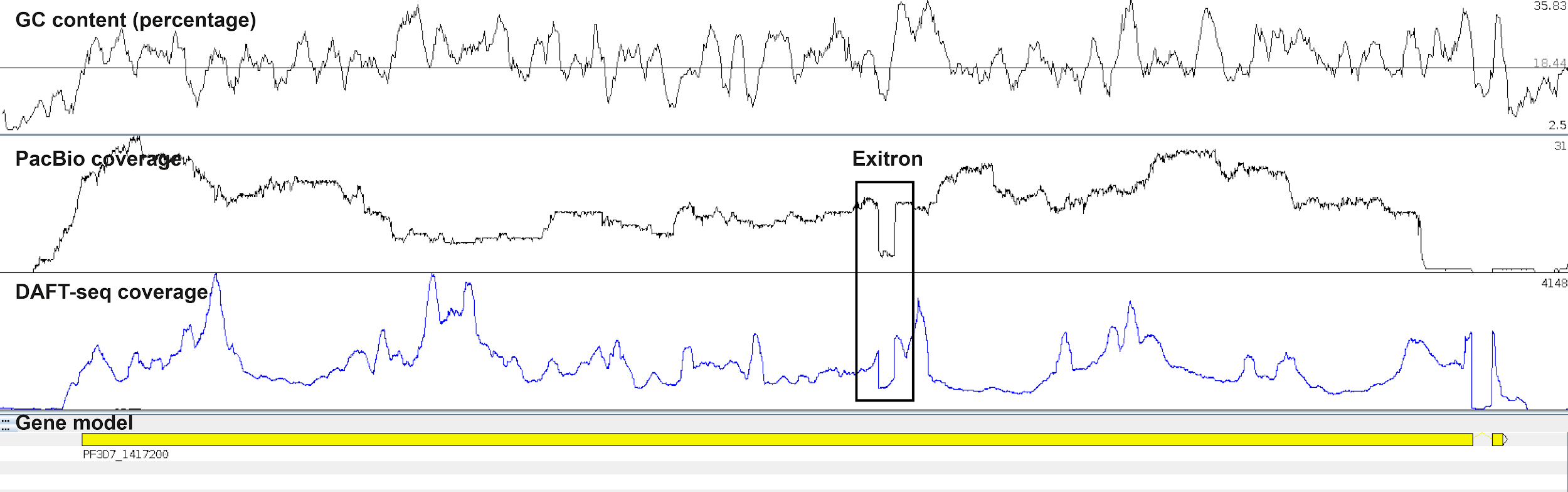
**

**Figure S22: Exitrons in PacBio reads**

PacBio coverage (top panel) and DAFT-seq coverage (middle panel) show the same splice site within the first protein-coding exon of the gene PF3D7_1417200, which can be identified as an exitron.

| 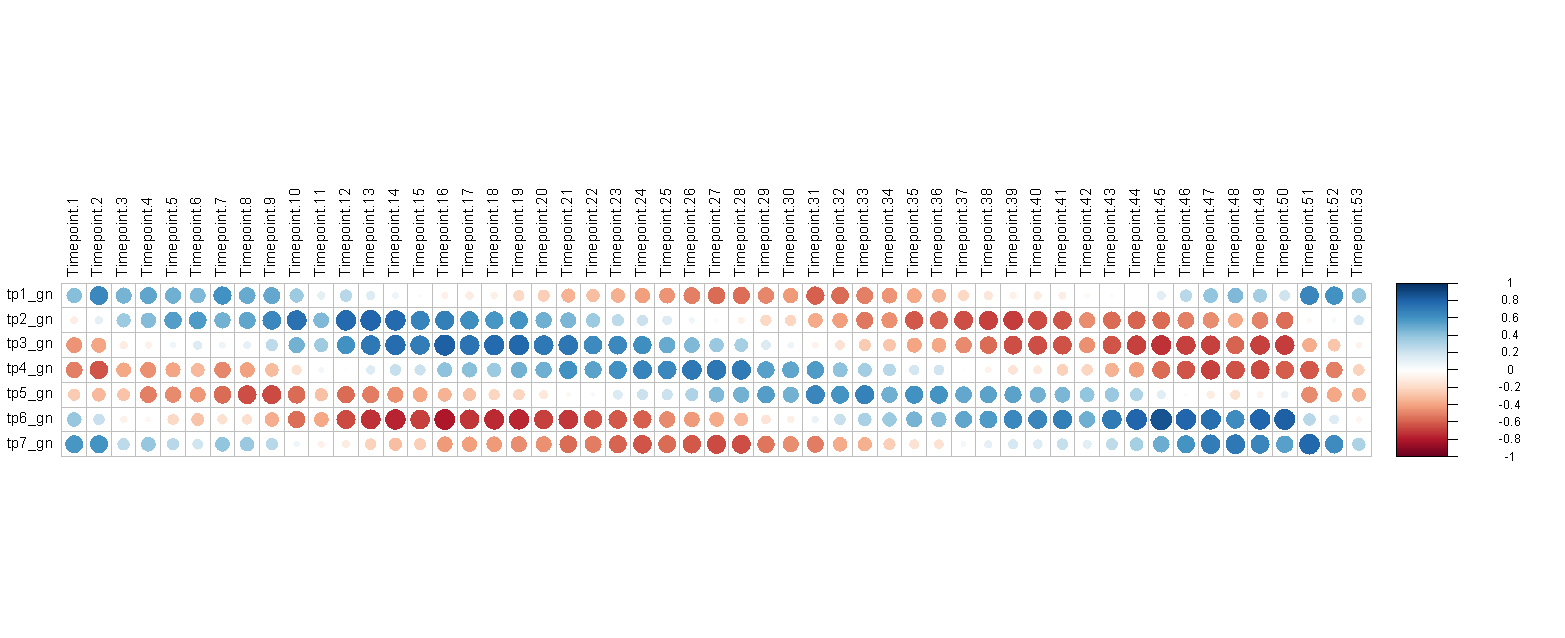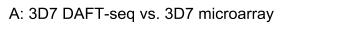 |
| --- |
| 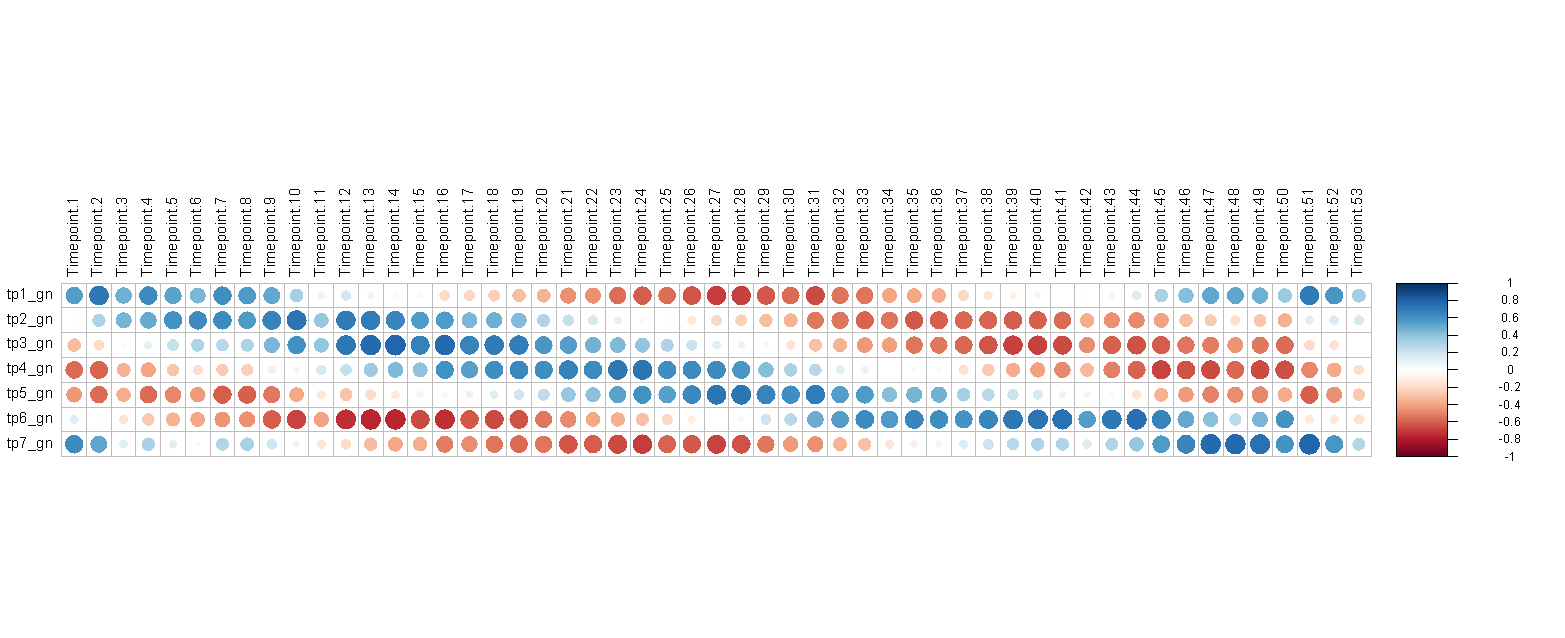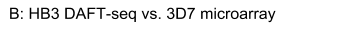 |
| 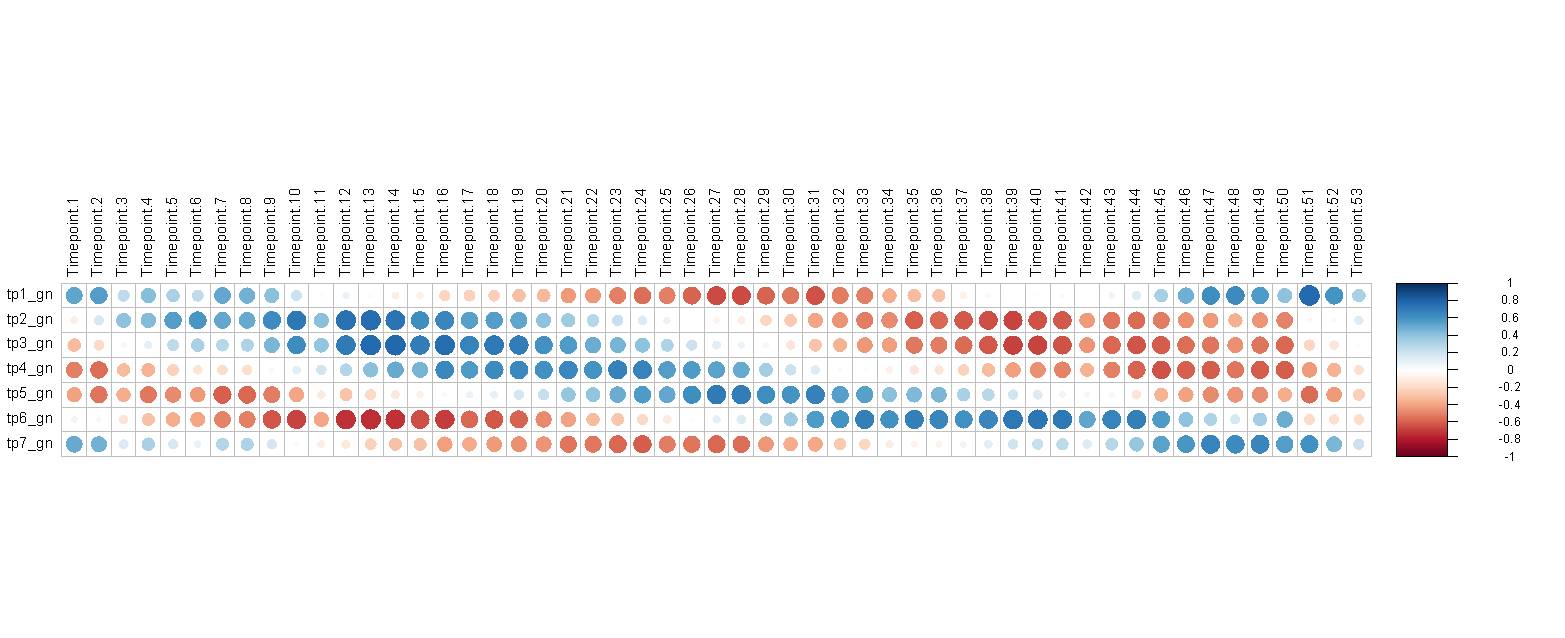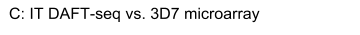 |

**Figure S23: Correlation of DAFT-seq data to existing 3D7 microarray data by correlation plots**

Horizontal data is from a published 3D7 array time course (Llinas et al., 2006). Data on the vertical axis is from the DAFT-seq time courses, which has been normalised to better match the format of the array data (see supplemental methods). High positive correlation between two time points is shown by large dark blue circles, with lighter smaller blue circles representing weaker positive correlation. Orange circles represent negative correlation. The trend observed is that the new DAFT-seq data best matches the data in expected time point from the array data set.

**
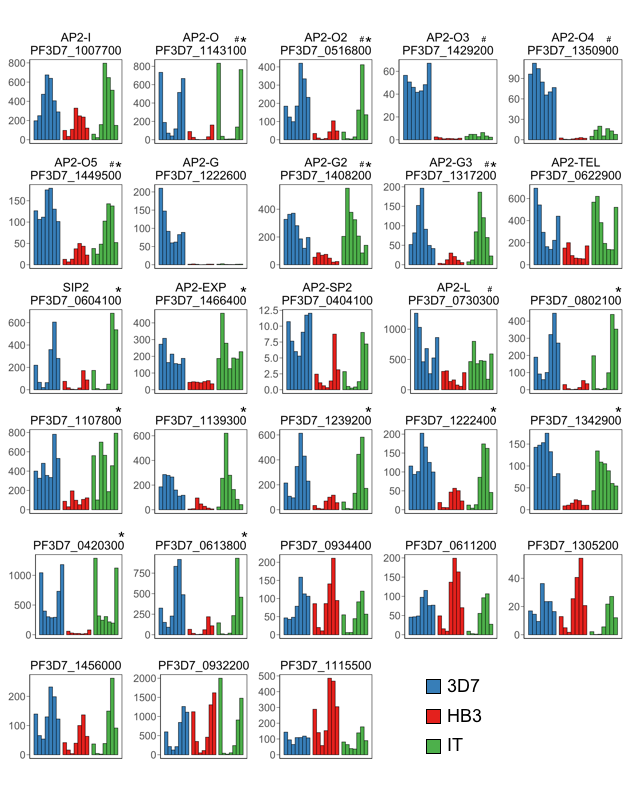
**

**Figure S24: ApiAP2 expression in the three *P. falciparum* strains**

Of 28 ApiAP2 transcription factors, as annotated based on the 3D7 genome on PlasmoDB, 15 are significantly overexpressed in 3D7 compared to HB3. Each plot shows the TPM calculated per time point, per strain per gene. Genes marked by an asterisk (*) are significantly overexpressed in 3D7 relative to HB3. Genes marked by a pound sign (#) represent those which have not yet been functionally validated in *P. falciparum*. All others were either not significantly different or transcription was undetected in one or both strains (TPM < 5).


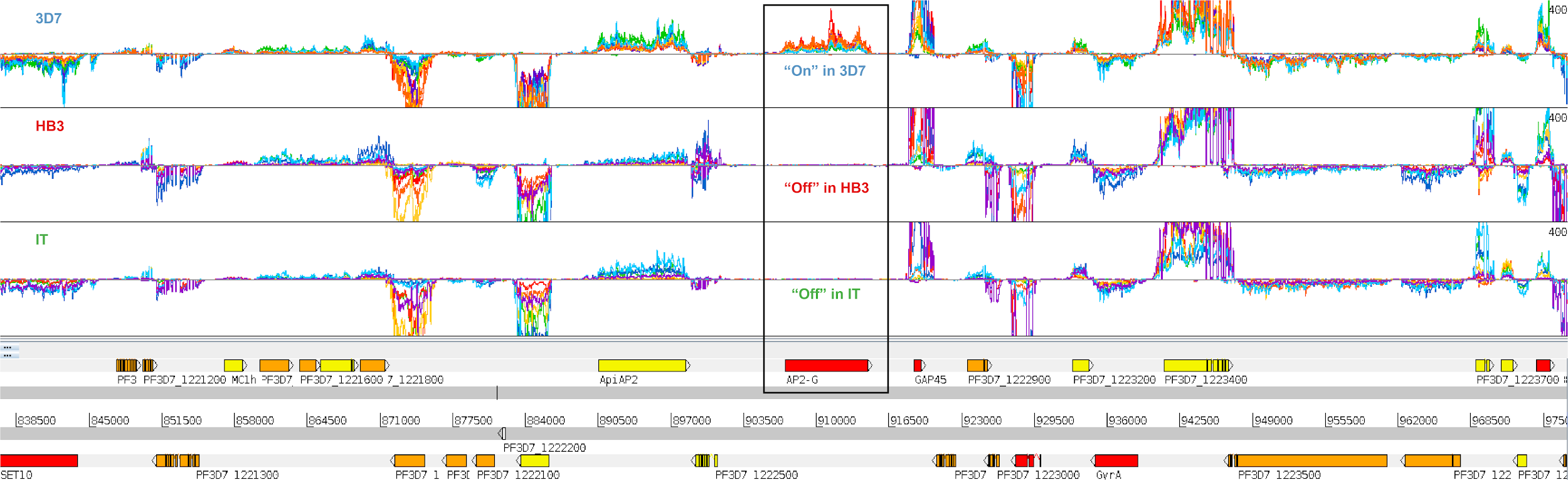


**Figure S25: AP2-G expression in the three *P. falciparum* strains**

Top panel: 3D7 DAFT-seq data; middle panel: HB3 DAFT-seq data; lower panel: IT DAFT-seq data; bottom panel: gene models (shown on both DNA strands). The seven time points are represented by the same colours in each panel (TP1=red, TP2=orange, TP3=yellow, TP4=green, TP5=light blue, TP6= dark blue, TP7=purple). Most genes show similar expression levels through the time course in each of the three strains, but the AP2-G gene is detected at higher steady state levels in the 3D7 strain (AP2-G is located within the black box).


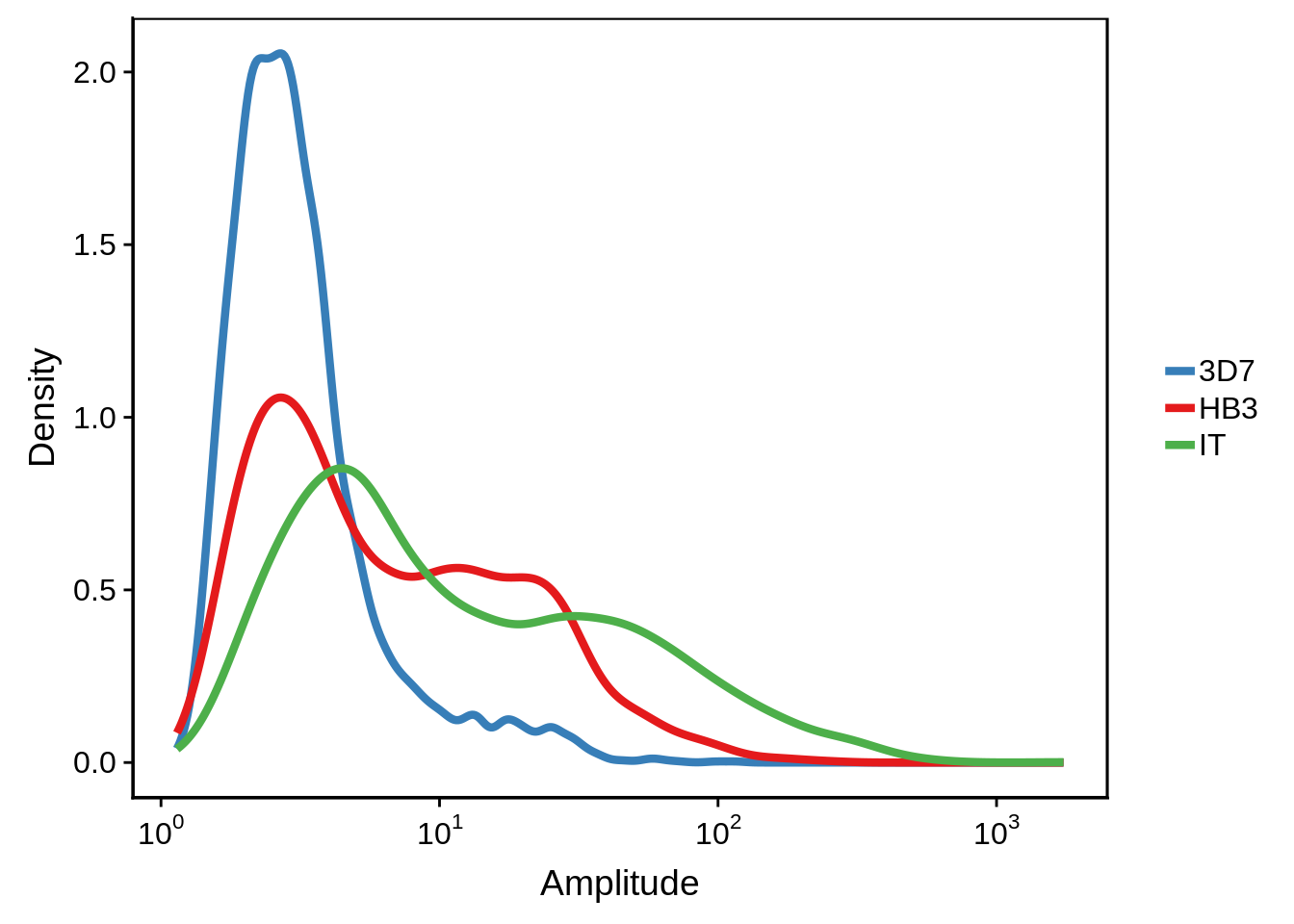


**Figure S26: Amplitude change distributions for all genes in the three *P. falciparum* strains**

Amplitudes for each gene were calculated as the difference between its maximum and minimum estimated TPM throughout the IDC.

| **3D7** | **HB3** | **IT** |
| --- | --- | --- |

**
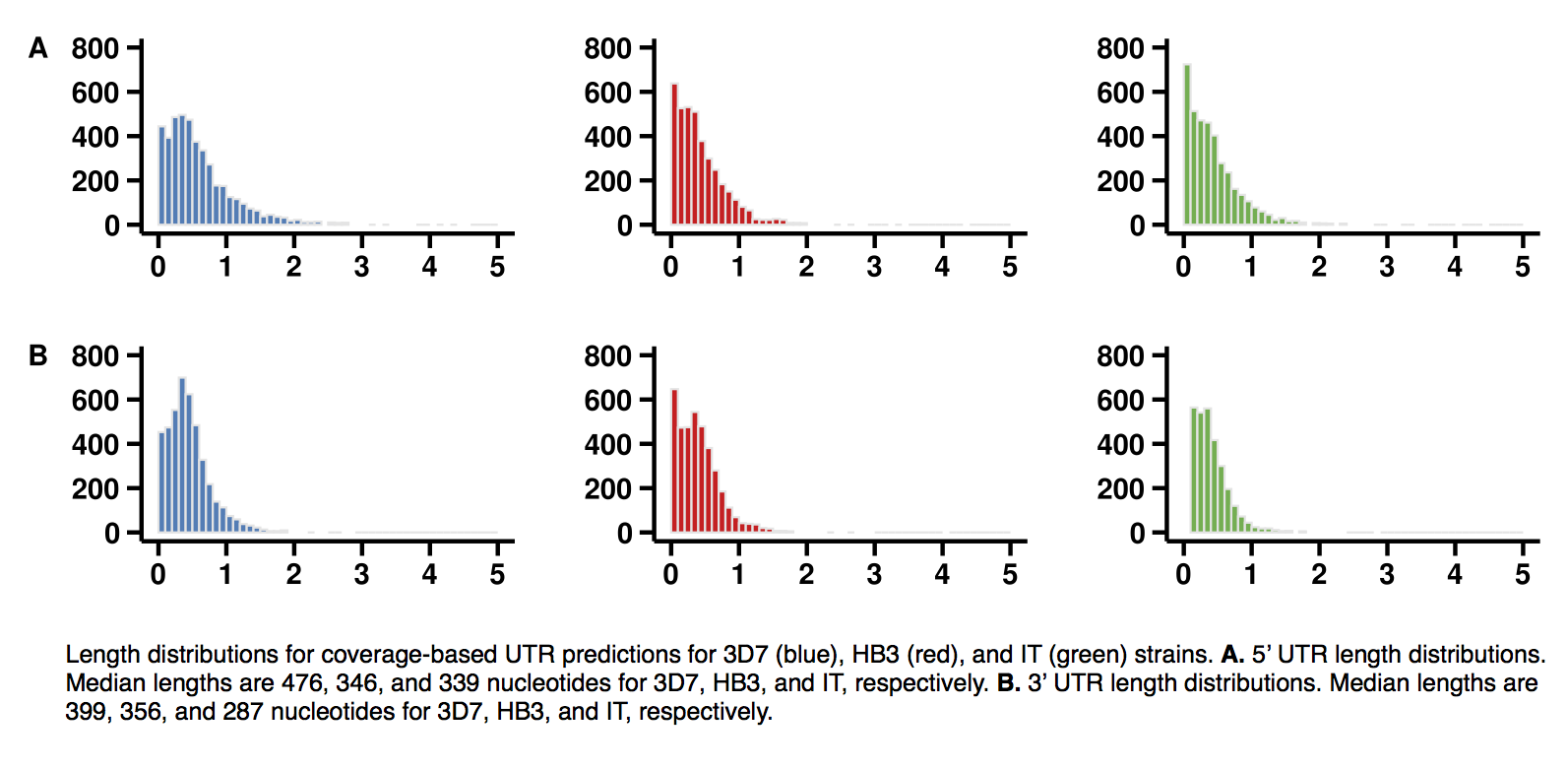
**

**Figure S27: Coverage-based UTRs for the three *P. falciparum* strains**

Length distributions for coverage-based UTR predictions for 3D7 (blue), HB3 (red), and IT (green) strains in kilobases (kb).

**A)** 5’ UTR length distributions. Median lengths are 476, 346, and 339, nucleotides, for 3D7, HB3, and IT, respectively.

**B)** 3’ UTR length distributions. Median lengths are 399, 356, and 287 nucleotides for 3D7, HB3, and IT, respectively.


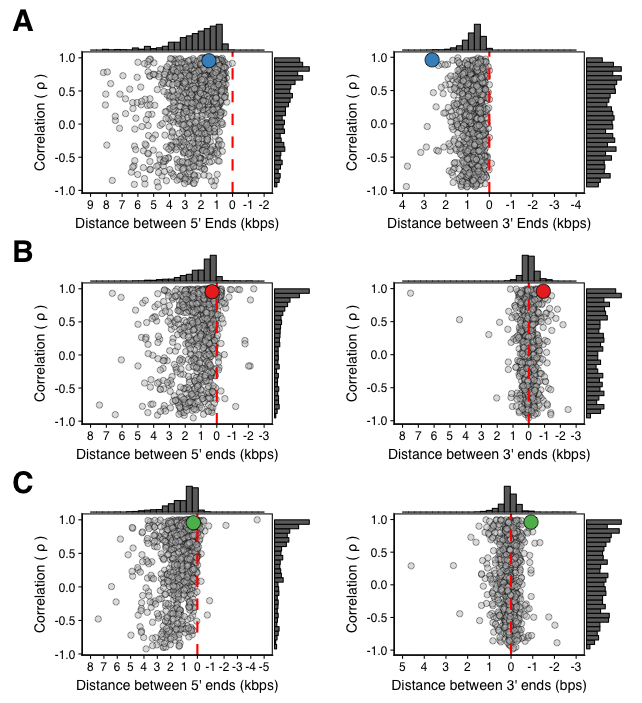


**Figure S28: Analysis of expression patterns of adjacent gene pairs for the three *P. falciparum* strains**

Correlation of gene expression (in TPM, using Pearson) for 1119 pairs of head-to-head genes and 1059 pairs of tail-to-tail genes. Large, colored data points represent the gene pair PF3D7_1011900 (heme oxygenase) and PF3D7_1012000 (putative RING zinc finger protein) in the left column and PF3D7_1115900 (DHHC9) and PF3D7_1116000 (RON4) in the right column.

**A)** Distance by correlation **without** annotated 5’ UTRs (left) or 3’ UTRs (right) plotted against the distance of intervening genomic sequence for the 3D7 strain. Without annotated 5’ UTRs, the median distance between head-to-head genes is 1946 bp. Without annotated 3’ UTRs, the median distance between tail-to-tail genes is 657 bp. (Note: figure 3 in main paper shows 3D7 with annotated UTRs)

**B)** Distance by correlation **with** annotated 5’ UTRs (left) or 3’ UTRs (right) plotted against the distance of intervening genomic sequence for the HB3 strain. The median distance between head-to-head and tail-to-tail genes is 713 and 23 bp, respectively.

**C)** Distance by correlation **with** annotated 5’ UTRs (left) or 3’ UTRs (right) plotted against the distance of intervening genomic sequence for the IT strain. The median distance between head-to-head and tail-to-tail genes is 762 and 70 bp, respectively.


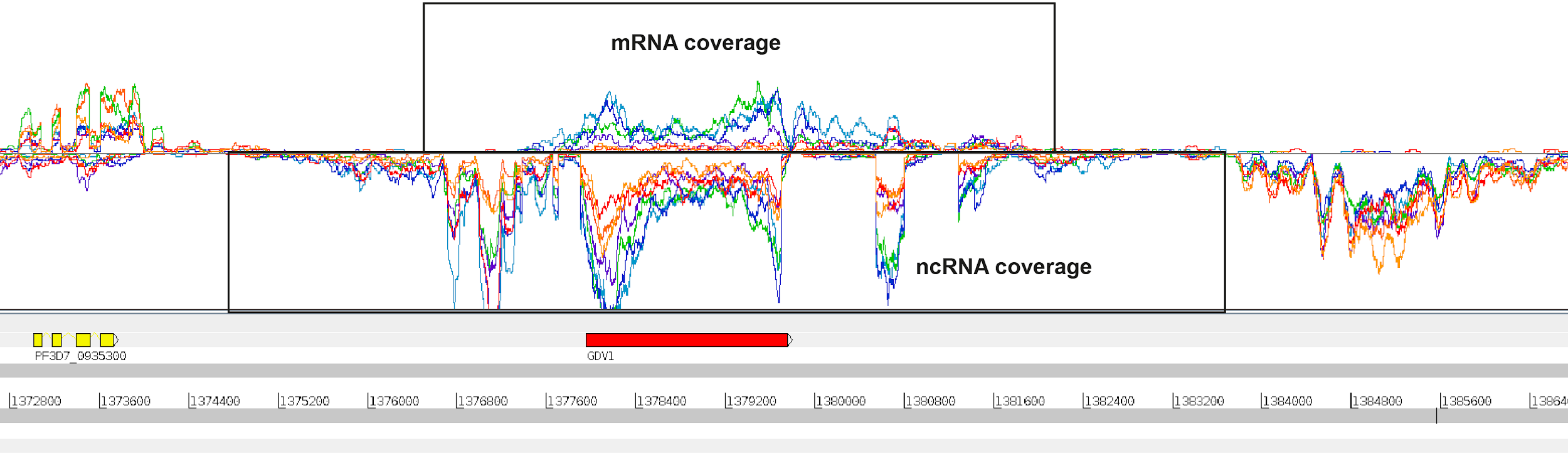


**Figure S29: A spliced non-coding RNA opposite GDV1**

A spliced non-coding RNA (marked within the region highlighted in the lower box) can be identified opposite the gene GDV1 (mRNA region marked within upper box) in the 3D7 DAFT-seq data.

**Supplementary text**

**Transcriptome realignments**

We used a multidimensional scaling (MDS) based approach to analyse phase differences of peak transcript levels between strains, similar to that in [[1]](https://paperpile.com/c/Fgrylf/9C8du), using the knowledge that many genes have a clear peak of expression in the IDC [[2]](https://paperpile.com/c/Fgrylf/sDR5T). This approach has been used in other model organisms such as yeast and humans when cyclic gene expression profiles were presumed [[3, 4]](https://paperpile.com/c/Fgrylf/BrqLf+OcrTO).

To apply MDS to our data, we first only included core, nuclear genes (i.e. non-telomeric as defined by GeneDB) that were transcribed (TPM of at least 5 within at least one sample) in all three strains. Additionally, only genes with an amplitude of at least 1.41 (2^0.5^) were included in our analysis, where the amplitude was calculated as the ratio of maximum to minimum TPM values throughout the time course, in order to filter out constitutively expressed or randomly fluctuating genes.

We then applied the isoMDS function found within the MASS package [[5]](https://paperpile.com/c/Fgrylf/lzC2l) to each time point for each strain and generate an uncorrected time point phase diagram as seen below:


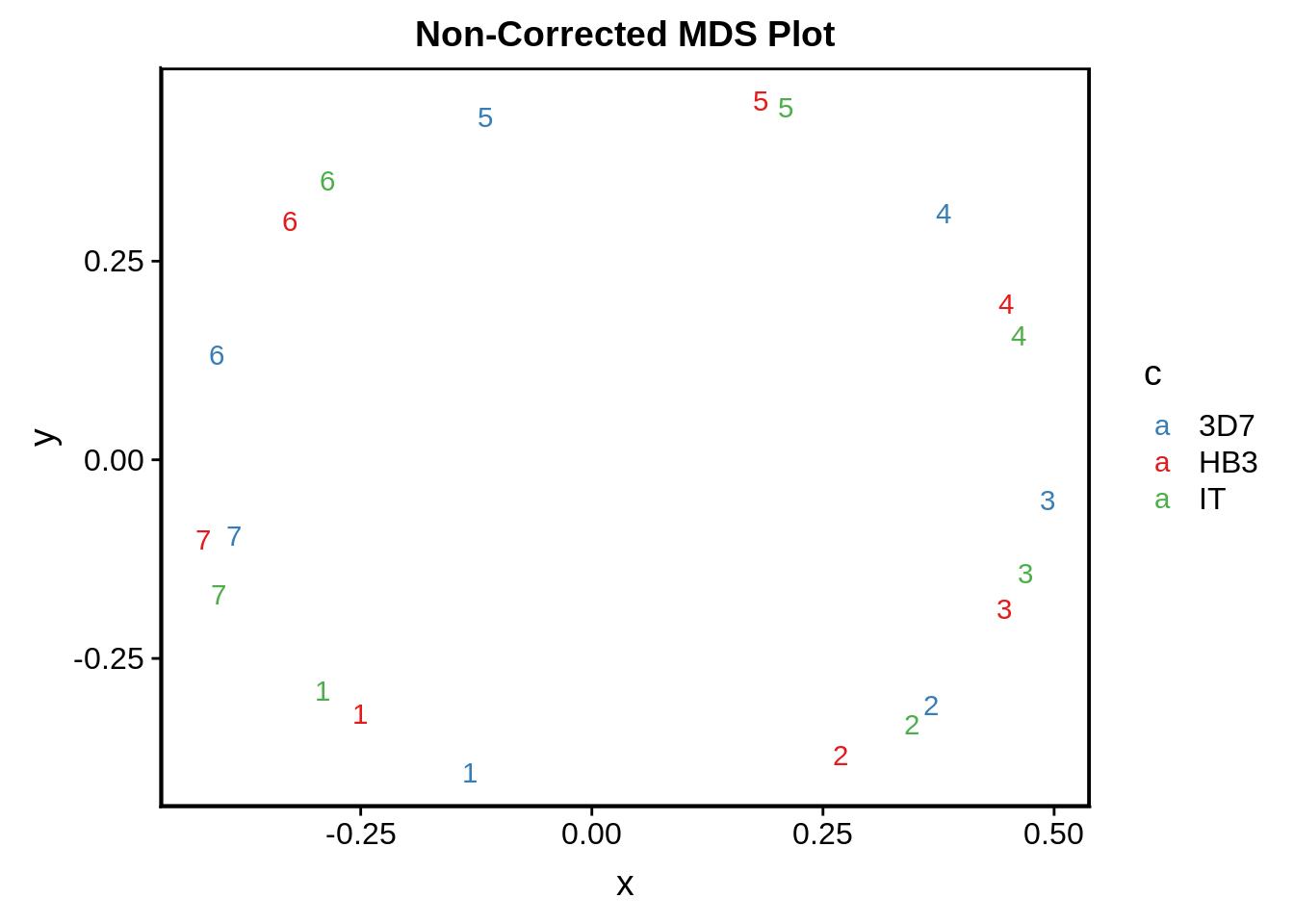


**Figure ST1: Non-Corrected MDS plot**

The colored numbers represent each of the seven time points in the three IDC time courses.

We observed that the 3D7 strain appears to be several hours “ahead” of the other strains..

We attempted to perform a local transcriptome alignment using the 3D7 53 time-point gene expression microarray from [[6]](https://paperpile.com/c/Fgrylf/YtA3s). The procedure was as follows:

1. TPMs were first mean normalized and standardized in order to compare to normalized microarray data. This involved defining the mean TPM value to zero. The mean and standard deviation are calculated across the time course for each gene, then subtracted from the mean for each TPM value, then divided by the standard deviation.
2. Expression values were then imputed for each hour within 48 hours for each time course by fitting a periodic spline (periodicSpline function of the splines R package, version 3.5.1, as periodic splines can interpolate a function which is periodic in nature) to the 7 observed time points, using a 48 hour cycle as an input parameter.
3. Next, a time point-by-time point correlation matrix was generated by correlating each time point within the imputed RNA-seq data set to the observed data in the 3D7 microarray data set.
4. The imputed time points within the RNA-seq data sets that were most highly correlated with a set of microarray time points(1, 8, 16, 24, 32, 40, and 48 hours) were selected. For example, if the most highly correlated time points could be 2, 7, 16, 23, 38, 47 for the imputed RNA-seq data sets. These were then interpreted as the corrected expression values for each gene at time points 1, 8, 16, 24, 32, 40, and 48.
5. Finally, another periodic spline is fit to the previous predictions in order to impute new values for the time course at hours 1, 8, 16, 24, 32, 40, and 48.
6. These expression values at time points 1, 8, 16, 24, 32, 40, and 48 were then used as input for a locally realigned MDS plot

**
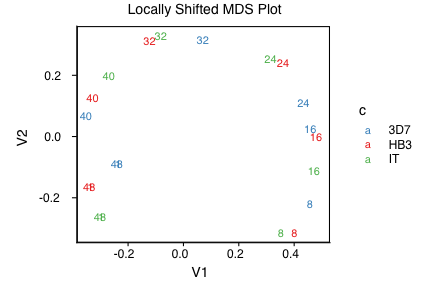
**

**Figure ST2: Locally shifted MDS plot**

The colored numbers represent each of the seven time points in the three IDC time courses.

We found that this method was able to successfully realign some, but not all time points. For example, time point 40 now appears to qualitatively cluster bettern, some time points did not. Most importantly, this did not change the overall phase difference histograms when comparing strains, suggesting that correcting for this experimental artifact is likely to be computationally infeasible with current tools. As a global realignment did not result in improved phase predictions, we decided to leave uncorrected, calculated phases as is for our main analysis of comparing the phases between strains.


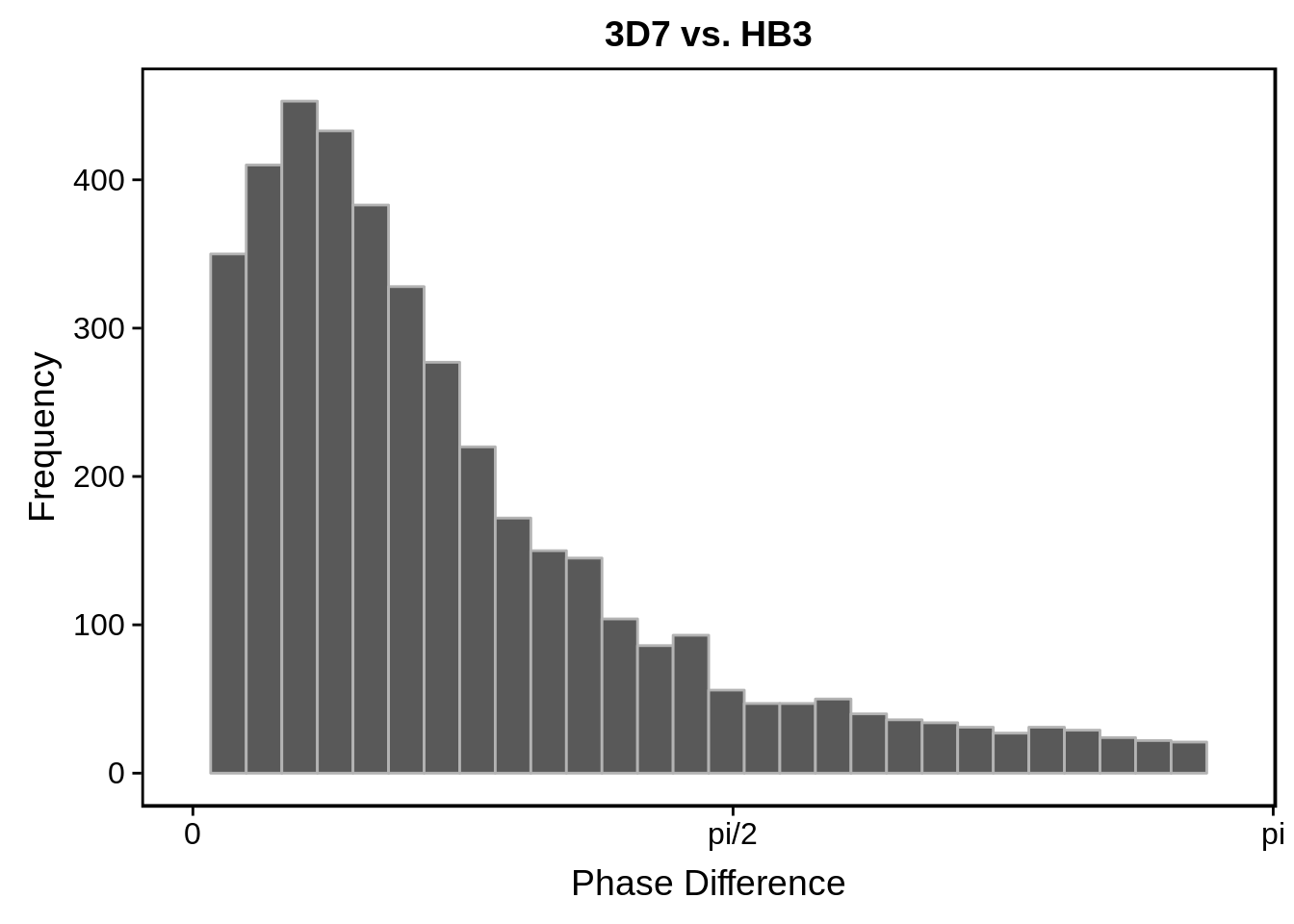

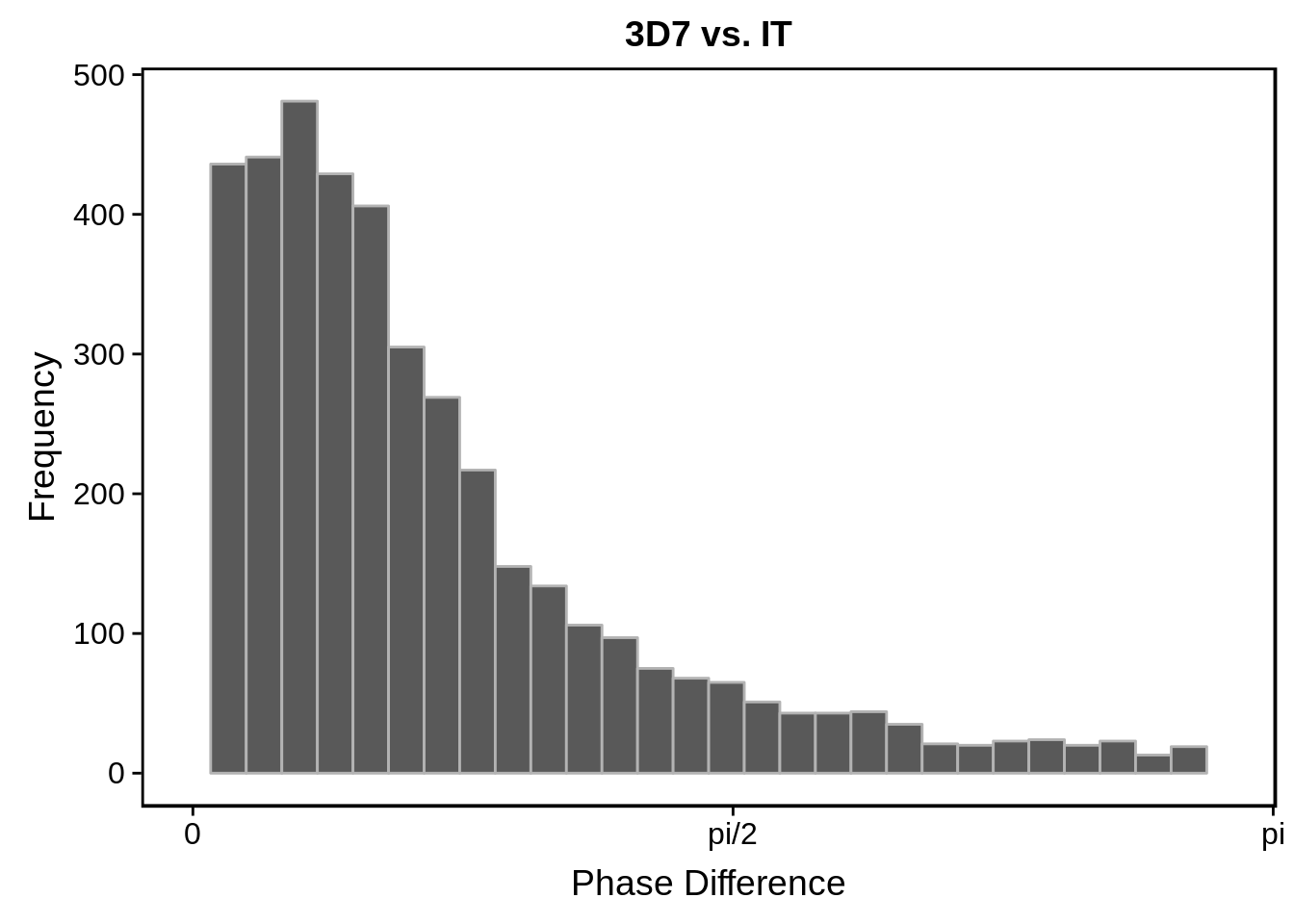

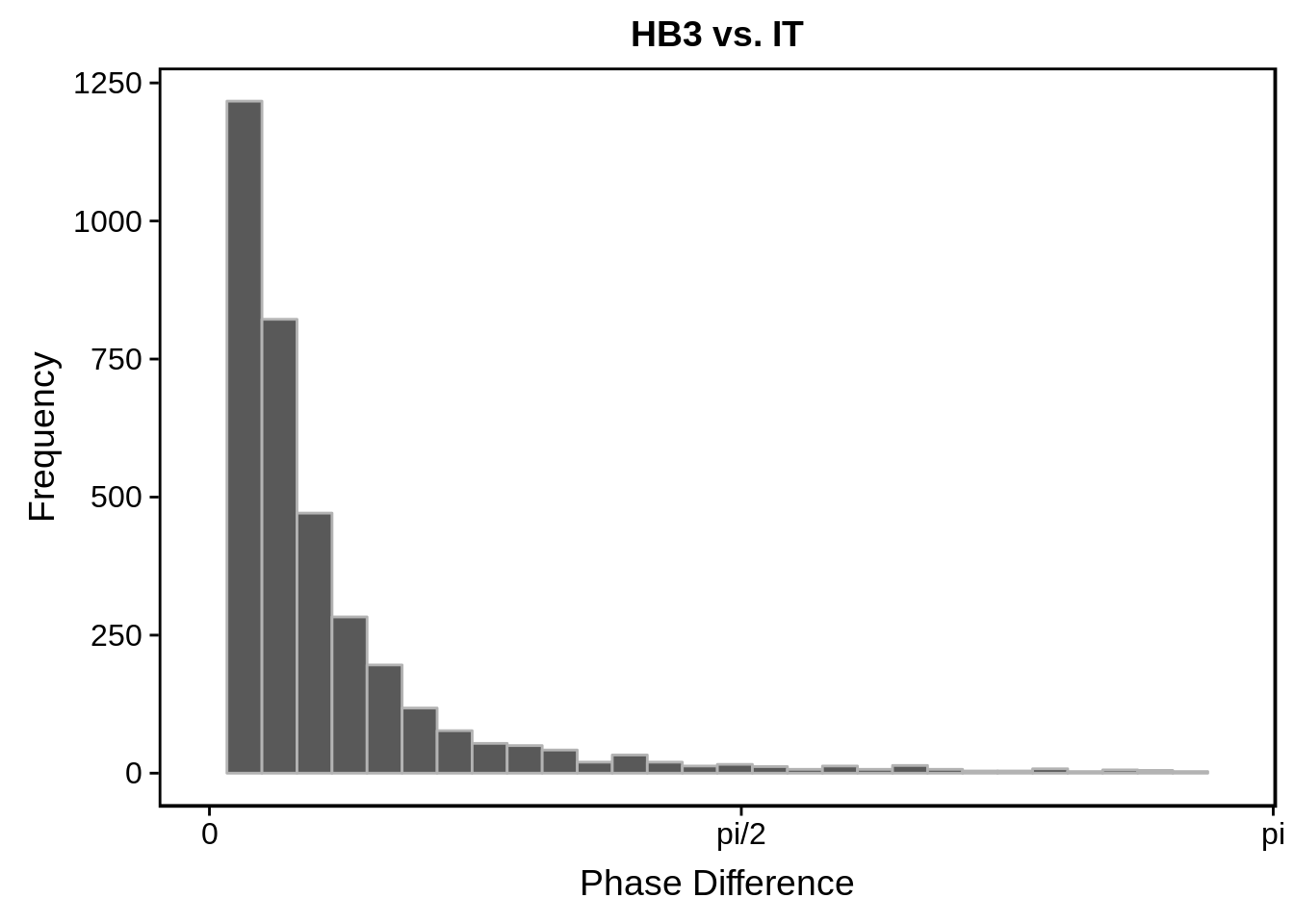


**Figure ST3: Phase differences for pairs of strains, using corrected data**

**UTR length comparisons**

We compared our UTR predictions to UTRs predicted in previous work (Figure S4, Figure S5). Caro *et al*. [[7]](https://paperpile.com/c/Fgrylf/9QanZ) predict 5’ and 3’ UTRs using RNA-seq data and a machine learning algorithm. However, they make these predictions for each time point in their time course. Similarly, Adjalley *et al.* [[8]](https://paperpile.com/c/Fgrylf/aAoeO) use a variant of CAGE sequencing [[9]](https://paperpile.com/c/Fgrylf/t0uOz) in order to define transcription start sites throughout the genome, for multiple time points.

For Caro *et al.*, the longest 5’ and 3’ UTR predictions for each gene were used to compare to our own. Pearson correlation estimates were generated using the cor.test function in R (method=”pearson”).

For Adjalley *et al.*, the longest, shortest, average, and median UTR lengths for each gene were calculated. These predictions were further filtered using a threshold cutoff of 4 as to compromise between conservative and liberal TSS predictions (see [[8]](https://paperpile.com/c/Fgrylf/aAoeO)) and a positive length (i.e. predicted transcription start sites cannot be downstream of annotated translation start sites). Finally, single nucleotide transcription start sites were determined by dividing the length of each “TSS block” by 2. That is, the TSS used in our comparison is the midpoint of each “TSS block.”

**Homopolymer tract analysis**

We wished to confirm why coverage-based UTRs sometimes suffered from sudden drops in read coverage within blocks of otherwise continuous transcription, as this was an artifact that can artificially shorten them. We suspected that homopolymer tracts were the cause of these coverage drops. Our ability to “rescue” missing fragments of coverage-based UTRs using the 5UTR-seq (TSO-based) TSSs is consistent with this hypothesis.

Key questions include:

- Do long homopolymer tracts exist within predicted coverage UTRs?
- Are UTRs containing long homopolymer tracts on average shorter than those that don’t?

To address these questions, we extended the 5’ UTRs 100 bp upstream beyond their predicted coverage-based TSS (i.e. prior to 5UTR-seq assisted correction), counted the length of the longest homopolymer tract for each nucleotide, and determined whether longer homopolymer tracts tend to occur in 5’ UTRs that are shorter. In order to account for the possibility that longer sequences will be more likely to contain longer homopolymer tracts by chance, the UTR lengths were normalized by the lengths of the longest homopolymer tracts found within each sequence. As is seen in Figure S9, we do find that shorter UTRs tend to have longer homopolymer tracts. Furthermore, if we focus only on UTRs that could be “repaired”, the correlation gets stronger and is strongest when we look only at 100 bp upstream of “repaired” 5’ UTRs. This suggests that 5’ UTRs in *P. falciparum*, using short-read technology, can only be effectively predicted using a combination of RNA-seq and TSS-seq.

**Analysis of TSS data with published genomics data sets**

For Figure S11 and Figure S12, reference-point enrichment plots for P. falciparum 3D7 transcription start sites were constructed for the following features: Nucleosome occupancy at 18 and 36 hours post infection [[10]](https://paperpile.com/c/Fgrylf/h5AW), H3K4Me3, H3K9ac, and H2A.Z at 40hpi [[11]](https://paperpile.com/c/Fgrylf/9aDv), H3K36Me2, H3K36Me3, and H3K9Me3 at 42 hours post infection [[12]](https://paperpile.com/c/Fgrylf/bV9A), Bromodomain Protein I in trophozoites [[13]](https://paperpile.com/c/Fgrylf/IEU3) and Heterochromatin Protein I in schizonts [[14]](https://paperpile.com/c/Fgrylf/04yj). Log2 normalized coverage data for the covalent histone markers H3K4Me3, H3K9ac, H3K9me3, H3K36Me2, and H3K36Me3 was downloaded from the repository https://github.com/Daread/plasmodium ExprPrediction hosted by the authors of Read et al. [[15]](https://paperpile.com/c/Fgrylf/nbII). All other datasets were downloaded from the SRA database and trimmed for adapter content using Trimmomatic [[16]](https://paperpile.com/c/Fgrylf/FUYz) then mapped to version 3 of the P. falciparum genome using bwa-mem [[17]](https://paperpile.com/c/Fgrylf/Axi4) using default parameters for paired/single end data as appropriate. Reads were filtered for duplicates and multiply mapped reads using samtools version 1.1 [[18]](https://paperpile.com/c/Fgrylf/MQXS) and log2 coverage of the immunoprecipitate over input was determined using bamCompare [[19]](https://paperpile.com/c/Fgrylf/i59c) with bin size 20, smooth length 60 and minimum mapping quality 30. Nucleosome occupancy relative to 1X coverage was determined using BamCoverage [[19]](https://paperpile.com/c/Fgrylf/i59c) with the same arguments plus the --mnase flag to account for mononucleosome size. These coverage tracks were plotted against the start of the 5' UTR with respect to strandedness for P. falciparum 3D7 such that the TSS = 5’UTR start from 5' to 3' using the DeepTools2 (version 3.4.1) ComputeMatrix and plotHeatmap functions [[19]](https://paperpile.com/c/Fgrylf/i59c). Genomic regions on the same chromosome and of equivalent size to the original 5’UTR were generated using shuffleBed from the BEDTools suite [[20]](https://paperpile.com/c/Fgrylf/kLvE) (version 2.17.0). The same chromatin enrichment datasets as above were plotted relative to these intervals as a negative control.

**Generating Position Weight Matrices**

Core motifs were identified as in Campbell *et al*. [[21]](https://paperpile.com/c/Fgrylf/0Ii1u). Full-length position weight matrices (PWMs) representing *in vitro* DNA-binding motifs for several ApiAP2 DNA-binding domains, were downloaded and manually converted to MEME format. PWMs were trimmed from the 5’ and 3’ ends to include only the six most informative positions (the core) of each PWM as calculated by the Shannon entropy.

**Expression of pseudogenes in DAFT-seq data**

We examined the expression of pseudogenes in the DAFT-seq data sets. Although the 3D7 genome is extremely well annotated and curated (see Boehme et al.[[22]](https://paperpile.com/c/Fgrylf/EKcsX); the authors of this paper used the data reported in our manuscript), the role or roles of these pseudogenes in gene function or regulation is not well understood. Interestingly, a recent manuscript[[23]](https://paperpile.com/c/Fgrylf/Qvs1U) has reported that a pseudogene is actually translated into a functional protein by *Plasmodium falciparum* parasites. Whether this holds true for other pseudogenes remains to be established.

We have manually inspected a number of annotated pseudogenes in the Artemis genome browser, and observe that there are transcripts present that are continuous and include both 5’ and 3’ UTRs, comparable to the annotated protein-coding genes. We suggest that many of these transcripts are present and “real”, and may be retained for regulatory purposes. One speculation is that the transcription of these genes must be retained in certain genomic locations, such as when a pseudogene is in a “head-to-head” orientation with an essential protein-coding gene. Experimental work outside the scope of our study would be necessary to investigate this further. The image below shows an image representing expression of the pseudogene EBA165 in our HB3 dataset, highlighted in red, which is head to head with the gene RH4. This observed expression of the EBA165 pseudogene was relevant for a study reported by our colleagues[[24]](https://paperpile.com/c/Fgrylf/YTlil) .

**
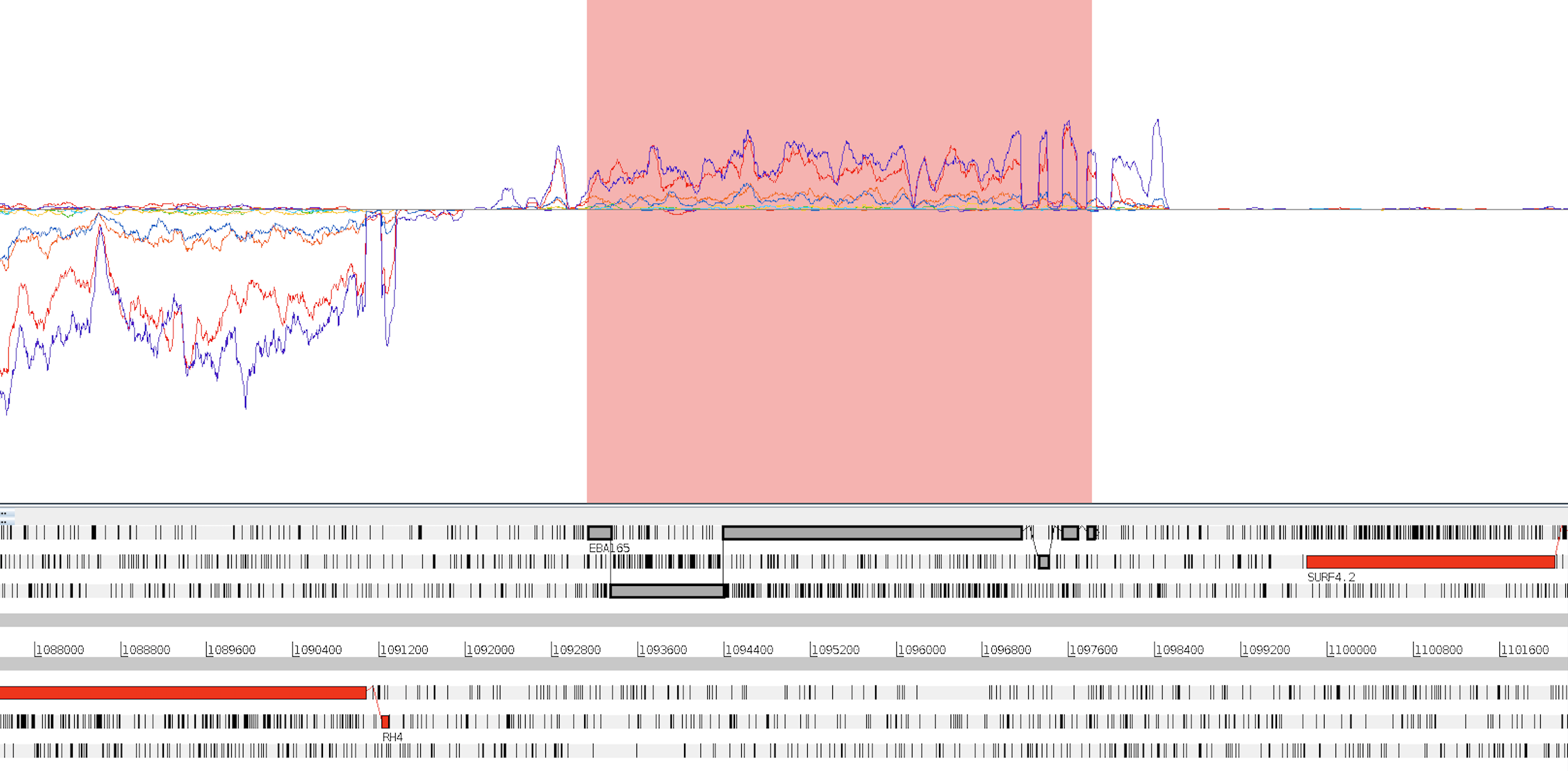
**

**Figure ST4: Transcription of the pseudogene EBA165, highlighted in red (data shown for the HB3 strain)**

We examined the expression of annotated pseudogenes in our datasets. There were 152 pseudogenes in our analysis, and 122 (80.2%) genes are transcribed in at least one of the three strains (TPM of at least 5 at one time point) and 62 (50.8%) were transcribed in all three strains. One interesting example from a recently posted preprint[[23]](https://paperpile.com/c/Fgrylf/Qvs1U), SURFIN 4.1 (PF3D7_0402200), shows high levels of transcription in 3D7, but much lower, although still detectable levels in HB3 and IT. We considered whether this high level of transcription was a consequence of bidirectional transcription, in light of previously published data that suggests that mutagenesis of this pseudogene is not essential for parasite survival, RH1 (PF3D7_0402300). Interestingly, the transcriptional profile of RH1 (PF3D7_0402300), the gene directly upstream in head-to-head orientation, matched the transcriptional profile of SURFIN 4.1 temporally, but the mRNA abundance of SURFIN 4.1 and RH1 between strains was very different. For SURFIN 4.1, transcript was detected at high levels only in 3D7, whereas RH1 transcription was detected at high levels in all three strains, with the highest occurring for IT. Thus, while bidirectional transcription may explain transcription of SURFIN 4.1 in 3D7, further experimentation will be required to determine why SURFIN 4.1 is not transcriptionally regulated in a similar bi-directional manner in HB3 or IT.

**
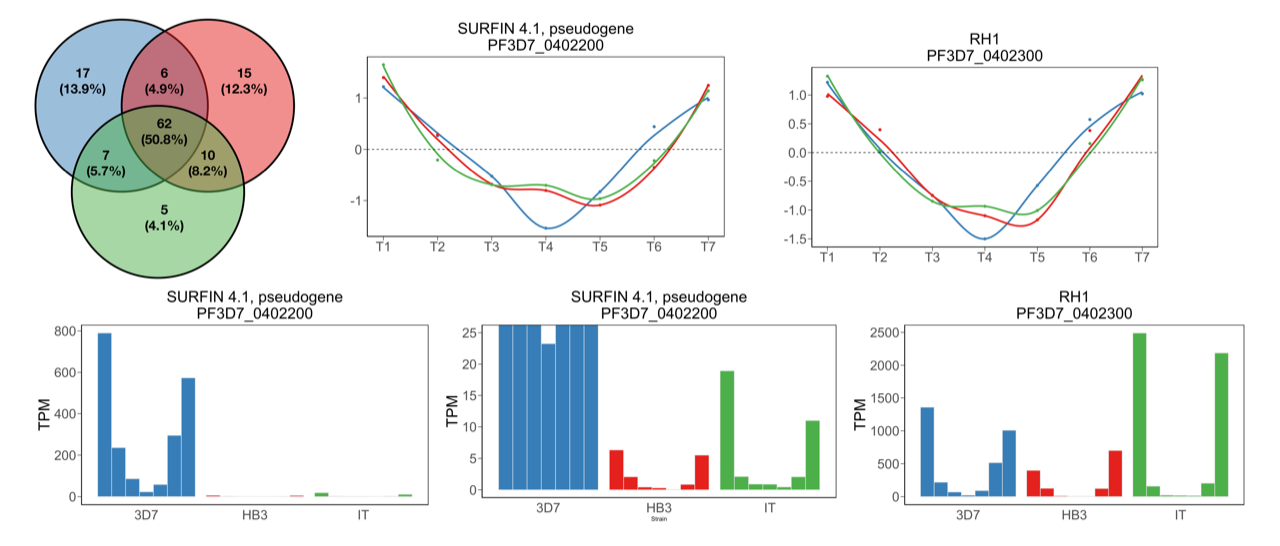
**

**Figure ST5: Expression patterns of pseudogenes in 3D7, HB3 and IT DAFT-seq data sets**

**References**

[1. Broadbent KM, Broadbent JC, Ribacke U, Wirth D, Rinn JL, Sabeti PC. Strand-specific RNA sequencing in Plasmodium falciparum malaria identifies developmentally regulated long non-coding RNA and circular RNA. BMC Genomics. 2015;16:454.](http://paperpile.com/b/Fgrylf/9C8du)

[2. Bozdech Z, Llinás M, Pulliam BL, Wong ED, Zhu J, DeRisi JL. The Transcriptome of the Intraerythrocytic Developmental Cycle of Plasmodium falciparum. PLoS Biology. 2003;1:e5. doi:](http://paperpile.com/b/Fgrylf/sDR5T)[10.1371/journal.pbio.0000005](http://dx.doi.org/10.1371/journal.pbio.0000005)[.](http://paperpile.com/b/Fgrylf/sDR5T)

[3. Rajaram S, Oono Y. NeatMap--non-clustering heat map alternatives in R. BMC Bioinformatics. 2010;11:45.](http://paperpile.com/b/Fgrylf/BrqLf)

[4. Tzeng J, Lu HH-S, Li W-H. Multidimensional scaling for large genomic data sets. BMC Bioinformatics. 2008;9:179.](http://paperpile.com/b/Fgrylf/OcrTO)

[5. Ripley B, Venables B, Bates DM, Hornik K, Gebhardt A, Firth D, et al. Package “mass.” CRAN Repos Httpcran R-Proj OrgwebpackagesMASSMASS Pdf. 2013.](http://paperpile.com/b/Fgrylf/lzC2l) <ftp://192.218.129.11/pub/CRAN/web/packages/MASS/MASS.pdf>[.](http://paperpile.com/b/Fgrylf/lzC2l)

[6. Llinás M, Bozdech Z, Wong ED, Adai AT, DeRisi JL. Comparative whole genome transcriptome analysis of three Plasmodium falciparum strains. Nucleic Acids Res. 2006;34:1166–73.](http://paperpile.com/b/Fgrylf/YtA3s)

[7. Caro F, Ahyong V, Betegon M, DeRisi JL. Genome-wide regulatory dynamics of translation in the Plasmodium falciparum asexual blood stages. Elife. 2014;3. doi:](http://paperpile.com/b/Fgrylf/9QanZ)[10.7554/eLife.04106](http://dx.doi.org/10.7554/eLife.04106)[.](http://paperpile.com/b/Fgrylf/9QanZ)

[8. Adjalley SH, Chabbert CD, Klaus B, Pelechano V, Steinmetz LM. Landscape and Dynamics of Transcription Initiation in the Malaria Parasite Plasmodium falciparum. Cell Rep. 2016;14:2463–75.](http://paperpile.com/b/Fgrylf/aAoeO)

[9. Takahashi H, Lassmann T, Murata M, Carninci P. 5′ end–centered expression profiling using cap-analysis gene expression and next-generation sequencing. Nature Protocols. 2012;7:542–61. doi:](http://paperpile.com/b/Fgrylf/t0uOz)[10.1038/nprot.2012.005](http://dx.doi.org/10.1038/nprot.2012.005)[.](http://paperpile.com/b/Fgrylf/t0uOz)

[10. Bunnik EM, Polishko A, Prudhomme J, Ponts N, Gill SS, Lonardi S, et al. DNA-encoded nucleosome occupancy is associated with transcription levels in the human malaria parasite Plasmodium falciparum. BMC Genomics. 2014;15:347.](http://paperpile.com/b/Fgrylf/h5AW)

[11. Bártfai R, Hoeijmakers WAM, Salcedo-Amaya AM, Smits AH, Janssen-Megens E, Kaan A, et al. H2A.Z Demarcates Intergenic Regions of the Plasmodium falciparum Epigenome That Are Dynamically Marked by H3K9ac and H3K4me3. PLoS Pathogens. 2010;6:e1001223. doi:](http://paperpile.com/b/Fgrylf/9aDv)[10.1371/journal.ppat.1001223](http://dx.doi.org/10.1371/journal.ppat.1001223)[.](http://paperpile.com/b/Fgrylf/9aDv)

[12. Jiang L, Mu J, Zhang Q, Ni T, Srinivasan P, Rayavara K, et al. PfSETvs methylation of histone H3K36 represses virulence genes in Plasmodium falciparum. Nature. 2013;499:223–7.](http://paperpile.com/b/Fgrylf/bV9A)

[13. Josling GA, Petter M, Oehring SC, Gupta AP, Dietz O, Wilson DW, et al. A Plasmodium Falciparum Bromodomain Protein Regulates Invasion Gene Expression. Cell Host Microbe. 2015;17:741–51.](http://paperpile.com/b/Fgrylf/IEU3)

[14. Fraschka SA, Filarsky M, Hoo R, Niederwieser I, Yam XY, Brancucci NMB, et al. Comparative Heterochromatin Profiling Reveals Conserved and Unique Epigenome Signatures Linked to Adaptation and Development of Malaria Parasites. Cell Host Microbe. 2018;23:407–20.e8.](http://paperpile.com/b/Fgrylf/04yj)

[15. Read DF, Cook K, Lu YY, Le Roch KG, Noble WS. Predicting gene expression in the human malaria parasite Plasmodium falciparum using histone modification, nucleosome positioning, and 3D localization features. PLoS Comput Biol. 2019;15:e1007329.](http://paperpile.com/b/Fgrylf/nbII)

[16. Bolger AM, Lohse M, Usadel B. Trimmomatic: a flexible trimmer for Illumina sequence data. Bioinformatics. 2014;30:2114–20.](http://paperpile.com/b/Fgrylf/FUYz)

[17. Li H, Durbin R. Fast and accurate short read alignment with Burrows-Wheeler transform. Bioinformatics. 2009;25:1754–60.](http://paperpile.com/b/Fgrylf/Axi4)

[18. Li H, Handsaker B, Wysoker A, Fennell T, Ruan J, Homer N, et al. The Sequence Alignment/Map format and SAMtools. Bioinformatics. 2009;25:2078–9.](http://paperpile.com/b/Fgrylf/MQXS)

[19. Ramírez F, Ryan DP, Grüning B, Bhardwaj V, Kilpert F, Richter AS, et al. deepTools2: a next generation web server for deep-sequencing data analysis. Nucleic Acids Res. 2016;44:W160–5.](http://paperpile.com/b/Fgrylf/i59c)

[20. Quinlan AR, Hall IM. BEDTools: a flexible suite of utilities for comparing genomic features. Bioinformatics. 2010;26:841–2.](http://paperpile.com/b/Fgrylf/kLvE)

[21. Campbell TL, De Silva EK, Olszewski KL, Elemento O, Llinás M. Identification and genome-wide prediction of DNA binding specificities for the ApiAP2 family of regulators from the malaria parasite. PLoS Pathog. 2010;6:e1001165.](http://paperpile.com/b/Fgrylf/0Ii1u)

[22. Böhme U, Otto TD, Sanders M, Newbold CI, Berriman M. Progression of the canonical reference malaria parasite genome from 2002-2019. Wellcome Open Res. 2019;4:58.](http://paperpile.com/b/Fgrylf/EKcsX)

[23. Macedo-Silva T, Araujo RBD, Wunderlich G. The pseudogene SURFIN 4.1 is vital for merozoite formation in blood stage P. falciparum. doi:](http://paperpile.com/b/Fgrylf/Qvs1U)[10.1101/562124](http://dx.doi.org/10.1101/562124)[.](http://paperpile.com/b/Fgrylf/Qvs1U)

[24. Proto WR, Siegel SV, Dankwa S, Liu W, Kemp A, Marsden S, et al. Adaptation of Plasmodium falciparum to humans involved the loss of an ape-specific erythrocyte invasion ligand. Nat Commun. 2019;10:4512.](http://paperpile.com/b/Fgrylf/YTlil)
